# Proteasome dysfunction underlies HERC2-linked neurodevelopmental disorder with Angelman-like clinical features

**DOI:** 10.1038/s41420-026-03095-x

**Published:** 2026-04-08

**Authors:** Joan Sala‑Gaston, Laura Costa‑Sastre, Manel Garcia‑Diez, Tania López‑Hernández, Juanma Ramírez, Nerea Osinalde, Jose Antonio Valer, Claudia Arnedo‑Pac, Bernat Crosas, Emma L. Baple, Andrew H. Crosby, Ugo Mayor, Francesc Ventura, Jose Luis Rosa

**Affiliations:** 1 Department of Physiological Sciences, University of Barcelona, IDIBELL, Barcelona, Spain; 2 Department of Biochemistry and Molecular Biology, Faculty of Science and Technology, UPV/EHU, Leioa, Spain; 3 Cambridge Institute for Medical Research (CIMR), University of Cambridge, Cambridge, UK; 4 MRC Toxicology Unit, University of Cambridge, Cambridge, UK; 5 IBMB‑CSIC, Barcelona, Spain; 6 University of Exeter Medical Research Centre, Exeter, UK; 7 Ikerbasque, Basque Foundation for Science, Bilbao, Spain

**Keywords:** Autism spectrum disorders, Protein-protein interaction networks, Ubiquitin ligases

## Abstract

Biallelic hypomorphic variants in the E3 ubiquitin ligase HERC2 cause a neurodevelopmental disorder clinically resembling Angelman syndrome, characterized by global developmental delay, intellectual disability, autism spectrum features, and movement abnormalities. Defining the target substrates of HERC2 is essential for understanding its biological role and the mechanisms underlying its pathological variants. To this end, we performed a quantitative proteomic analysis using biotinylated ubiquitin to identify HERC2 targets and assess their regulation in cells expressing HERC2 with or without ubiquitin‑ligase activity. This approach revealed extensive sets of subunits from major multimeric complexes, including the proteasome, the tRNA–biosynthesis machinery, microtubule‑associated assemblies, vesicle‑coat complexes, centrosomes, and the Ski and GATOR2 complexes, as substrates of HERC2-dependent ubiquitylation. Among these, the proteasome emerged as the most prominently affected complex. We identified up to eleven proteins required for assembly of the 19S regulatory particle whose ubiquitylation depends on HERC2. Mechanistically, we show that HERC2 recognizes unassembled subunits via chaperone-mediated interactions and targets them for proteasomal degradation. Loss of this mechanism in HERC2‑deficient cells alters proteasomal activity. It is noteworthy that fibroblasts derived from patients carrying the common pathogenic variant c.1781 C > T (p.Pro594Leu) exhibit impaired processing of 19S subunits and an aberrant increase in proteasome activity. Our findings establish a link between HERC2-related neurodevelopmental disorder and impaired proteasome activity, elucidate the molecular mechanisms through which HERC2 regulates proteostasis and how its disruption contributes to human pathology, and underscore potential therapeutic strategies for affected individuals.

## Introduction

Pathologenic variants in the E3 ubiquitin ligases HERC1 and HERC2 have been implicated in distinct neurodevelopmental disorders. Biallelic *HERC1* variants cause *Macrocephaly, Dysmorphic Facies, and PsychoMotor Retardation* (MDFPMR) syndrome (OMIM #617011) [1, 2]. Likewise, biallelic hypomorphic variants in HERC2 result in a neurodevelopmental disorder that clinically resembles Angelman syndrome, featuring global developmental delay, intellectual disability, autism spectrum characteristics, and movement abnormalities (OMIM #615516) [2–8].

A key step toward understanding the biological functions of these ligases and the mechanisms by which their pathogenic variants exert their effects is to define their target substrates. Using a biotinylated ubiquitin (^bio^Ub) approach combined with comparative quantitative proteomics [9], we identified a broad set of subunits from multimeric complexes whose ubiquitylation is regulated by HERC2. Interaction assays, functional studies, and analyses of patient‑derived fibroblasts collectively reveal a central role for HERC2 in proteasome assembly and activity.

## Results

### ^Bio^Ub-based proteomics uncovers HERC2-dependent ubiquitylation networks

**Fig. 1:**
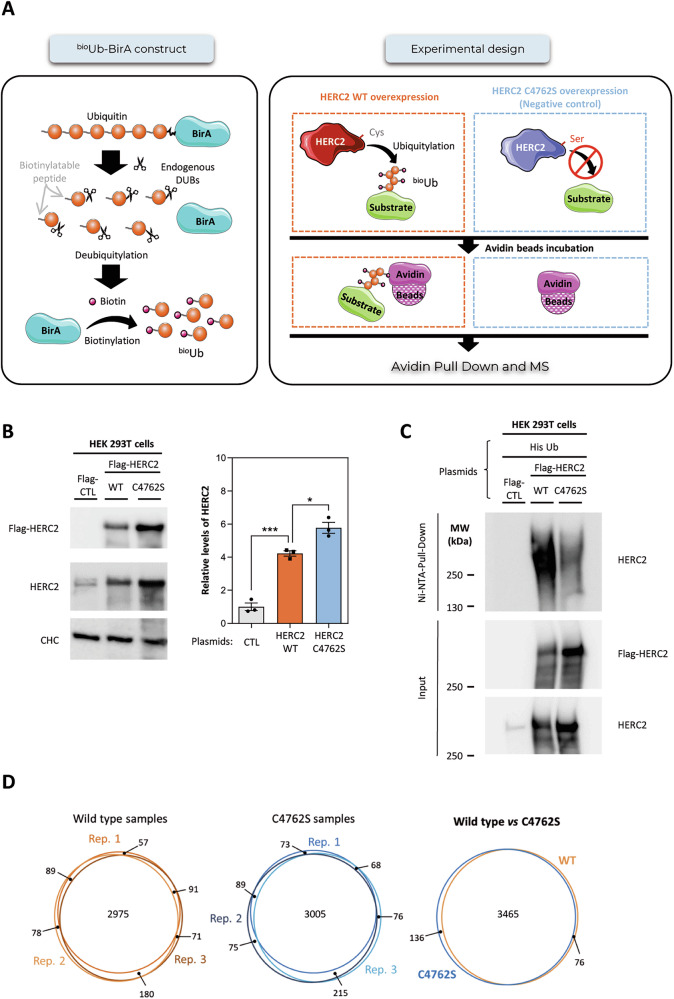
^Bio^Ub-based proteomics identifies protein ubiquitylation regulated by HERC2. **A** The ^bio^Ub strategy is based on the in vivo biotinylation of ubiquitin (Ub) achieved by transfecting the ^bio^Ub-BirA construct, and combined with the overexpression of the wild-type HERC2 (HERC2 WT) or a mutant HERC2 (HERC2 C4762S) without ubiquitin ligase activity. Ubiquitylated proteins are isolated with avidin beads and identified by mass spectrometry (MS). **B** HEK-293T cells were transfected with a negative control plasmid (Flag-CTL), a plasmid encoding wild-type HERC2 protein fused with Flag epitope (Flag-HERC2 WT) or a plasmid encoding a catalytically inactive form (Flag-HERC2 C4762S). The indicated proteins were analyzed by immunoblot. The results are expressed relative to the control condition. Plots represent mean ± standard error of the mean. Representative results are shown from experiments (*n* = 3), and the individual data points are plotted as single dots. Significance levels: * *p* ≤ 0.05; *** *p* ≤ 0.001. **C** HEK-293T cells were transfected with the indicated plasmids. After 48 h, cells were incubated for 6 h with MG132 (10 µM). Ubiquitylated proteins were purified using a Ni-NTA-agarose resin. Inputs and pull-downs were analyzed by immunoblotting with antibodies against the indicated proteins. Representative results are shown. **D** Overlap between proteins identified in HERC2 wild type (depicted in orange) and HERC2 C4762S samples (depicted in blue). Venn diagrams showing the overlap of the proteins identified among the three replicas of each condition, as well as between both conditions, are shown.

To identify HERC2 ubiquitylation substrates, we performed the ^bio^Ub strategy [9], combining gene overexpression and proteomics analysis. This ^bio^Ub strategy is based on the in vivo biotinylation of ubiquitin achieved by transfecting the ^bio^Ub-BirA construct (Fig. 1A). This construct encodes a precursor polypeptide composed of six ubiquitin molecules, which had been modified in their N-terminus with a short biotinylable peptide. In addition, the biotin ligase enzyme of *E.Coli* BirA is fused at the C-terminus of the precursor peptide. Once the ^bio^Ub-BirA construct is expressed in cells, it is digested by endogenous deubiquitinases, releasing ubiquitin molecules and enabling BirA to label them with biotin in its N-terminus. This strategy is combined with the overexpression of the wild-type HERC2 (HERC2 WT) and a mutant HERC2 (HERC2 C4762S) version without ubiquitin ligase activity. Ubiquitylated substrates are then isolated using avidin beads and subsequently identified by mass spectrometry. By directly comparing samples from HERC2 WT and the catalytically inactive HERC2 C4762S variant, this approach enables the precise identification of ubiquitylated proteins that depend on HERC2 activity (Fig. 1A).

Lysates from transfected HEK-293T cells were first analyzed by western blot, and results showed a significant overexpression of HERC2 proteins, which were significantly higher than the mutated form (Fig. 1B). One possible explanation is that HERC2 regulates its own ubiquitylation-mediated proteasome degradation. To verify this, an ubiquitylation assay was performed using His-tagged ubiquitin. Ubiquitylated proteins were pulled-down and analyzed by SDS-PAGE and immunoblotting. The smear of high molecular weight, indicating HERC2 ubiquitylation, was much greater in HERC2 WT. These results demonstrate that HERC2 regulates its own ubiquitylation (Fig. 1C).

**Fig. 2:**
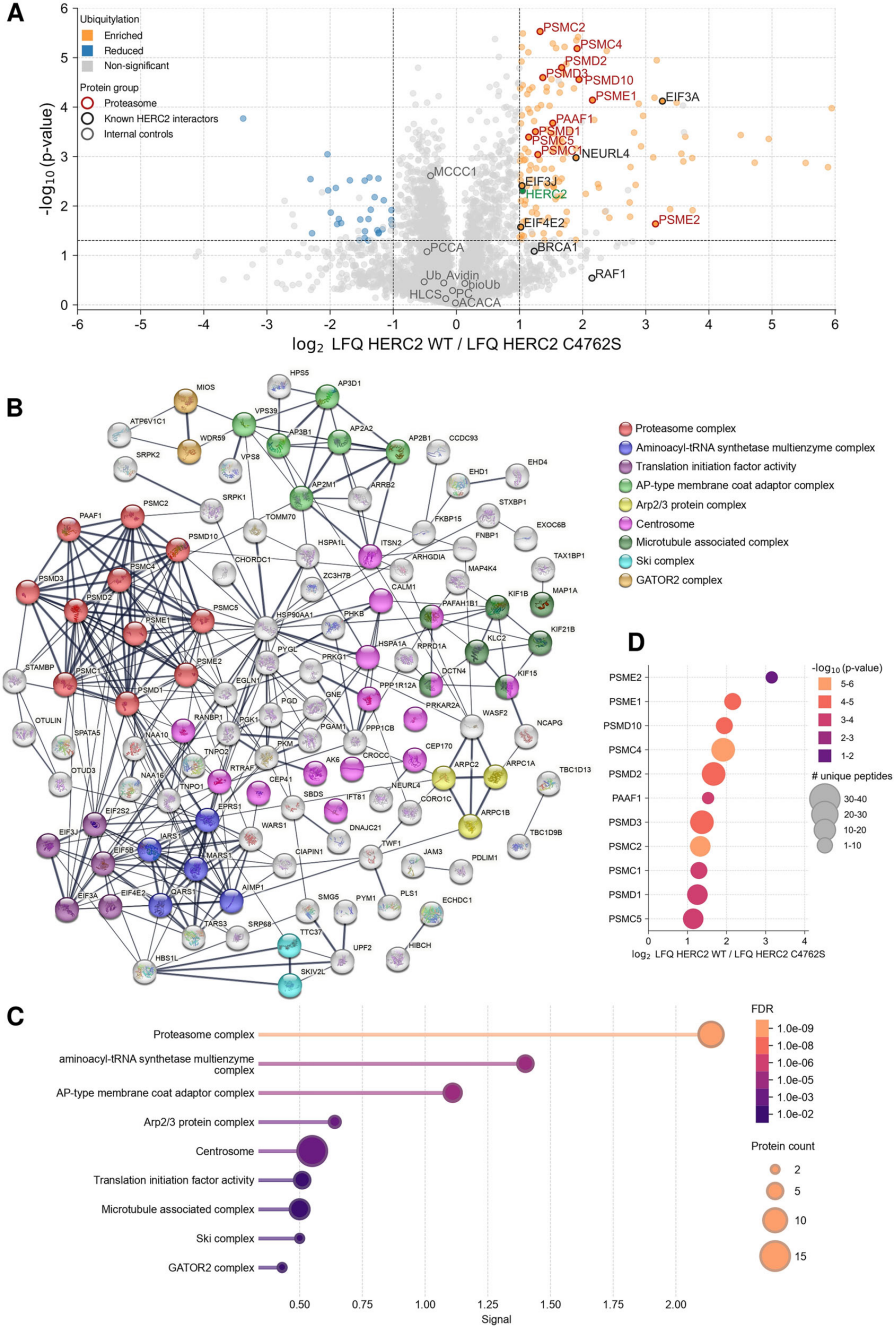
Identification of ubiquitylated proteins regulated by HERC2 and their functional connectivity. **A** Volcano plot shows differentially ubiquitylated proteins upon HERC2 WT overexpression relative to HERC2 C4762S. Abundance of each individual protein was determined by its label-free quantification (LFQ) intensities. The LFQ WT/C4762S ratios (log_2_ scale) and the t-test p-values (−log_10_ scale) are displayed in the X and Y axes, respectively. The threshold for statistical significance (*p*-value < 0.05) is indicated with a horizontal gray line, while vertical gray lines depict a > 1 log_2_ fold increase or decrease of the ubiquitylated levels upon HERC2 WT overexpression. Proteins that pass these thresholds, and fulfill the quality control criteria, and show a significant increase or reduction of their ubiquitination levels upon HERC2 WT overexpression are shown in orange and blue, respectively (log_2_ LFQ WT/LFQ C4762S > 1 marked in orange; log_2_ LFQ WT/LFQ C4762S < -1 marked in blue). Previously described or suggested substrates are shown in black. HERC2 is shown in green, and endogenously biotinylated proteins (ACACA, MCCC1, PC, PCCA, HLCS), ubiquitin (Ub), ^bio^Ub, and avidin (AVD) are shown in gray. Proteasome-related proteins are shown in red. **B** Functional protein association network using STRING shows functional connectivity of ubiquitylated proteins regulated by HERC2. **C** Functional enrichment analysis of the protein clusters detailed in (**B**). **D** Detailed significance, fold change and number of unique detected peptides by MS for each proteasome-related complex protein identified in (**A**, **B**).

Triplicate samples of HERC2 WT and HERC2 C4762S-overexpressing HEK-293T cells were subjected to proteomic analysis using ^bio^Ub strategy. A high reproducibility across the biological replicas was observed (Fig. 1D) (Supplementary Fig. 1). A total of 3677 proteins were identified, of which 3465 were found both in wild-type and C4762S samples (Fig. 1D). Of these, 139 increased, and 31 decreased in ubiquitylation with HERC2 WT vs C4762S (Fig. 2A and Tables 1; 2). To further characterize the 139 ubiquitylated proteins, we used STRING (Search Tool for the Retrieval of Interacting Genes/Proteins) to evaluate functional protein association networks and hence identify functional enrichments of any biological or cellular function. The analysis highlighted nine major connectivity clusters: proteasome complex, aminoacyl-tRNA synthetase multienzyme complex, translation initiation factor activity, AP-type membrane coat adaptor complex, Arp2/3 protein complex, centrosome, microtubule-associated complex, Ski complex, and GATOR2 complex (Fig. 2B, C). In addition to these identified complexes and clusters, 74/139 ubiquitylated proteins were identified to be part of a protein-containing complex (GO:0032991) as a stable set of interacting proteins. All these significant connectivity clusters were sorted by their signal, highlighting the proteasome complex as the most relevant cluster (Fig. 2C).

**Table 1:** Ubiquitylated proteins up-regulated by HERC2.

Gene names	Protein names	^a^FC
*PHKB*	Phosphorylase b kinase regulatory subunit beta	5.95
*VPS8*	Vacuolar protein sorting-associated protein 8 homolog	5.89
*LUZP1*	Leucine zipper protein 1	5.53
*PPP1R12A*	Protein phosphatase 1 regulatory subunit 12A	4.95
*STXBP1*	Syntaxin-binding protein 1	4.73
*SRPK2*	SRSF protein kinase 2; SRSF protein kinase 2 N-terminal; SRSF protein kinase 2 C-terminal	4.50
*WASF2*	Wiskott-Aldrich syndrome protein family member 2	3.74
*CORO1C*	Coronin-1C	3.74
*TARSL2 (TARS3)*	Threonine--tRNA ligase 2; Threonyl-tRNA synthetase 3	3.63
*UPF2*	Regulator of nonsense transcripts 2	3.60
*GGA1*	ADP-ribosylation factor-binding protein GGA1	3.57
*SPATA5*	Spermatogenesis-associated protein 5	3.49
*MTMR3*	Myotubularin-related protein 3	3.39
*EIF3A*	Eukaryotic translation initiation factor 3 subunit A	3.26
*PGAM1*	Phosphoglycerate mutase 1	3.17
*PSME2*	Proteasome activator complex subunit 2	3.15
*JAM3*	Junctional adhesion molecule C	3.14
*MAP1A*	Microtubule-associated protein 1A; MAP1A heavy chain; MAP1 light chain LC2	3.12
*GCC1*	GRIP and coiled-coil domain-containing protein 1	2.96
*PDLIM1*	PDZ and LIM domain protein 1	2.93
*CEP41*	Centrosomal protein of 41 kDa	2.89
*PPP1CB*	Serine/threonine-protein phosphatase PP1-beta catalytic subunit	2.84
*MIOS*	WD repeat-containing protein mio	2.82
*CRYZ*	Quinone oxidoreductase	2.76
*DNAJC21*	DnaJ homolog subfamily C member 21	2.75
*HIBCH*	3-hydroxyisobutyryl-CoA hydrolase, mitochondrial	2.75
*CALM*	Calmodulin-1	2.42
*EPRS (EPRS1)*	Bifunctional glutamate/proline--tRNA ligase;Glutamate--tRNA ligase;Proline--tRNA ligase	2.38
*VPS39*	Vam6/Vps39-like protein	2.36
*MSTO1*	Protein misato homolog 1	2.26
*WIBG (PYM1)*	Partner of Y14 and mago	2.24
*TBC1D13*	TBC1 domain family member 13	2.18
*PSME1*	Proteasome activator complex subunit 1	2.16
*OTUD3*	OTU domain-containing protein 3	2.05
*PSMD10*	26S proteasome non-ATPase regulatory subunit 10	1.94
*HSP90AA1*	Heat shock protein HSP 90-alpha	1.93
*PSMC4*	26S protease regulatory subunit 6B	1.91
*MARS (MARS1)*	Methionine--tRNA ligase, cytoplasmic	1.90
*NEURL4*	Neuralized-like protein 4	1.90
*EHD1*	EH domain-containing protein 1	1.89
*PROSC (PLPBP)*	Pyridoxal phosphate homeostasis protein	1.89
*C14orf166 (RTRAF)*	RNA transcription, translation and transport factor protein	1.86
*EHD4*	EH domain-containing protein 4	1.82
*AK6*	Adenylate kinase isoenzyme 6	1.75
*FKBP15*	FK506-binding protein 15	1.75
*QARS (QARS1)*	Glutamine--tRNA ligase	1.74
*TBC1D9B*	TBC1 domain family member 9B	1.74
*ARPC1A*	Actin-related protein 2/3 complex subunit 1 A	1.71
*C12orf4*	Protein C12orf4	1.71
*GNE*	Bifunctional UDP-N-acetylglucosamine 2-epimerase/N-acetylmannosamine kinase;UDP-N-acetylglucosamine 2-epimerase (hydrolyzing);N-acetylmannosamine kinase	1.71
*IBTK*	Inhibitor of Bruton tyrosine kinase	1.69
*EIF5B*	Eukaryotic translation initiation factor 5B	1.68
*NT5DC1*	5-nucleotidase domain-containing protein 1	1.67
*PSMD2*	26S proteasome non-ATPase regulatory subunit 2	1.67
*KLC2*	Kinesin light chain 2	1.67
*PYGL*	Glycogen phosphorylase, liver form	1.66
*FNBP1*	Formin-binding protein 1	1.66
*PKM*	Pyruvate kinase PKM	1.65
*SRPK1*	SRSF protein kinase 1	1.64
*NAA10*	N-alpha-acetyltransferase 10	1.63
*IFT81*	Intraflagellar transport protein 81 homolog	1.63
*RANBP1*	Ran-specific GTPase-activating protein	1.60
*TNPO1*	Transportin-1	1.59
*CCDC93*	Coiled-coil domain-containing protein 93	1.58
*AP2M1*	AP-2 complex subunit mu	1.57
*ARRB2*	Beta-arrestin-2	1.57
*HSP90AB4P*	Putative heat shock protein HSP 90-beta 4	1.55
*PLS1*	Plastin-1	1.54
*NAA16*	N-alpha-acetyltransferase 16, NatA auxiliary subunit	1.54
*EIF2S2*	Eukaryotic translation initiation factor 2 subunit 2	1.54
*IARS (IARS1)*	Isoleucine--tRNA ligase, cytoplasmic	1.54
*TTC37*	Tetratricopeptide repeat protein 37	1.53
*PAAF1*	Proteasomal ATPase-associated factor 1	1.53
*CHORDC1*	Cysteine and histidine-rich domain-containing protein 1	1.51
*NAP1L1*	Nucleosome assembly protein 1-like 1	1.51
*RNF214*	RING finger protein 214	1.51
*PAFAH1B1*	Platelet-activating factor acetylhydrolase IB subunit alpha	1.50
*ARHGDIA*	Rho GDP-dissociation inhibitor 1	1.48
*KIF1B*	Kinesin-like protein KIF1B	1.46
*WDR59*	WD repeat-containing protein 59	1.44
*RPRD1A*	Regulation of nuclear pre-mRNA domain-containing protein 1A	1.44
*ATP6V1C1*	V-type proton ATPase subunit C 1	1.43
*SRP68*	Signal recognition particle subunit SRP68	1.43
*ARPC1B*	Actin-related protein 2/3 complex subunit 1B	1.43
*ITSN2*	Intersectin-2	1.39
*OTULIN*	Ubiquitin thioesterase otulin	1.39
*HPS5*	Hermansky-Pudlak syndrome 5 protein	1.38
*PSMD3*	26S proteasome non-ATPase regulatory subunit 3	1.37
*STAMBP*	STAM-binding protein	1.35
*ANXA6*	Annexin A6	1.34
*PSMC2*	26S protease regulatory subunit 7	1.33
*WARS(WARS1)*	Tryptophan--tRNA ligase, cytoplasmic;T1-TrpRS;T2-TrpRS	1.32
*PSMC1*	26S protease regulatory subunit 4	1.29
*HSPA1B;HSPA1A*	Heat shock 70 kDa protein 1B;Heat shock 70 kDa protein 1 A	1.28
*TWF1*	Twinfilin-1	1.28
*ARPC2*	Actin-related protein 2/3 complex subunit 2	1.26
*KIF15*	Kinesin-like protein KIF15	1.26
*ARHGAP5*	Rho GTPase-activating protein 5	1.26
*PSMD1*	26S proteasome non-ATPase regulatory subunit 1	1.25
*CPNE3*	Copine-3	1.23
*HSPA1L*	Heat shock 70 kDa protein 1-like	1.20
*C11orf54*	Ester hydrolase C11orf54	1.20
*TAX1BP1*	Tax1-binding protein 1	1.20
*KIF21B*	Kinesin-like protein KIF21B	1.19
*SKIV2L*	Helicase SKI2W	1.18
*SBDS*	Ribosome maturation protein SBDS	1.17
*AP3B1*	AP-3 complex subunit beta-1	1.16
*CIAPIN1*	Anamorsin	1.16
*PSMC5*	26S protease regulatory subunit 8	1.15
*DCTN4*	Dynactin subunit 4	1.14
*MDH1*	Malate dehydrogenase, cytoplasmic	1.14
*AASDHPPT*	L-aminoadipate-semialdehyde dehydrogenase-phosphopantetheinyl transferase	1.14
*PGD*	6-phosphogluconate dehydrogenase, decarboxylating	1.12
*SMG5*	Protein SMG5	1.12
*MAP4K4*	Mitogen-activated protein kinase kinase kinase kinase 4	1.10
*WDR11*	WD repeat-containing protein 11	1.10
*TNPO2*	Transportin-2	1.09
*SEPTIN8*	Septin-8	1.09
*AP3D1*	AP-3 complex subunit delta-1	1.08
*PRKG1*	cGMP-dependent protein kinase 1	1.07
*PBDC1*	Protein PBDC1	1.07
*SCRN3*	Secernin-3	1.06
*EXOC6B*	Exocyst complex component 6B	1.05
*AP2B1*	AP-2 complex subunit beta	1.05
*HERC2*	E3 ubiquitin-protein ligase HERC2	1.05
*EIF3J*	Eukaryotic translation initiation factor 3 subunit J	1.04
*TOMM70A (TOMM70)*	Mitochondrial import receptor subunit TOM70	1.04
*AP2A2*	AP-2 complex subunit alpha-2	1.04
*EGLN1*	Egl nine homolog 1	1.03
*PGK1*	Phosphoglycerate kinase 1	1.03
*CROCC*	Rootletin	1.03
*ECHDC1*	Ethylmalonyl-CoA decarboxylase	1.02
*PRKAR2A*	cAMP-dependent protein kinase type II-alpha regulatory subunit	1.02
*CEP170*	Centrosomal protein of 170 kDa	1.02
*EIF4E2*	Eukaryotic translation initiation factor 4E type 2	1.02
*HBS1L*	HBS1-like protein	1.02
*AIMP1*	Aminoacyl tRNA synthase complex-interacting multifunctional protein 1; Endothelial monocyte-activating polypeptide 2	1.01
*NCAPG*	Condensin complex subunit 3	1.01
*ZC3H7B*	Zinc finger CCCH domain-containing protein 7B	1.01
*FAIM*	Fas apoptotic inhibitory molecule 1	1.00

^a^FC: LFQ HERC2 WT/LFQ HERC2 C4762S in log_2_ scale.

**Table 2:** Ubiquitylated proteins are down-regulated by HERC2.

Gene names	Protein names	^a^FC
*P4HA1*	Prolyl 4-hydroxylase subunit alpha-1	–3.38
*BRD2*	Bromodomain-containing protein 2	–2.31
*LMAN1*	Protein ERGIC-53	–2.29
*RER1*	Protein RER1	–2.04
*WIZ*	Protein Wiz	–2.03
*SFT2D2*	Vesicle transport protein SFT2B	–1.99
*YLPM1*	YLP motif-containing protein 1	–1.91
*TJP2*	Tight junction protein ZO-2	–1.89
*AATF*	Protein AATF	–1.86
*LAMB1*	Laminin subunit beta-1	–1.82
*BRD4*	Bromodomain-containing protein 4	–1.69
*RNH1*	Ribonuclease inhibitor	–1.54
*WBP11*	WW domain-binding protein 11	–1.52
*SPRYD3*	SPRY domain-containing protein 3	–1.45
*PPP6R2*	Serine/threonine-protein phosphatase 6 regulatory subunit 2	–1.45
*FLNC*	Filamin-C	–1.45
*RAB9A;RAB9B*	Ras-related protein Rab-9A;Ras-related protein Rab-9B	–1.40
*RLIM*	E3 ubiquitin-protein ligase RLIM	–1.39
*SON*	Protein SON	–1.38
*RPN2*	Dolichyl-diphosphooligosaccharide--protein glycosyltransferase subunit 2	–1.36
*CLDN12*	Claudin-12	–1.29
*NEFL*	Neurofilament light polypeptide	–1.25
*KIAA0922*	Transmembrane protein 131-like	–1.23
*DCPS*	m7GpppX diphosphatase	–1.23
*ZFR*	Zinc finger RNA-binding protein	–1.23
*KIF20B*	Kinesin-like protein KIF20B	–1.17
*APLP2*	Amyloid-like protein 2	–1.15
*RAB39B*	Ras-related protein Rab-39B	–1.03
*SUN1*	SUN domain-containing protein 1	–1.03
*NCCRP1*	F-box only protein 50	–1.02
*ITGAV*	Integrin alpha-V; Integrin alpha-V heavy chain; Integrin alpha-V light chain	–1.01

^a^FC: LFQ HERC2 WT/LFQ HERC2 C4762S in log_2_ scale.

### HERC2 promotes the ubiquitylation of proteins involved in the proteasome assembly machinery

The proteasome is a large protein complex with protease activity responsible for degrading proteins that are misfolded, damaged by stress conditions, or targeted for degradation as part of regular protein turnover. The most abundant proteasome in mammals is the 30S/26S proteasome, which is comprised of a 20S core particle (CP) and 19S regulatory particle (RP). While the 20S CP acts as the catalytic component of the 26S proteasome, the 19S RP acts as a proteasomal activator, being responsible for substrate recognition, deubiquitylation, protein unfolding and transferring to the 20S CP [10]. 19S RP is composed of two substructures: the base and lid subcomplexes. The base comprises a heterohexameric ring of six ATPases (PSMC1-6). The ATPases subunits are required not only for substrate unfolding with the energy liberated from hydrolysis of ATP, but also for α-ring channel opening. Additionally, other non-ATPase proteins are also part of the base subcomplex (PSMD1, PSMD2, PSMD4 and ADRM1). PSMD2, PSMD4 and ADRM1 recognize ubiquitylated proteins, while PSMD1 facilitates stabilization of the complex. The lid comprises nine proteins (PSMD3, PSMD6, PSMD7, PSMD8, PSMD11, PSMD12, PSMD13, PSMD14 and SEM1), which are involved in the recruitment of ubiquitylated proteins, deubiquitylation of substrates and stabilizing the proteasome. The base and the lid assemble in an independent way, with the action of different chaperones (PSMD5, PSMD9, PSMD10, and PAAF1) being crucial. HERC2 increased the ubiquitylation of eleven proteasome-related proteins (Fig. 2A, B, D), including components of the base (PSMC1, PSMC2, PSMC4, PSMC5, PSMD1, PSMD2), lid (PSMD3), and 19S assembly chaperones (PSMD10, PAAF1). This pattern suggests that HERC2 contributes to 19S assembly and, consequently, to proteasome activity [10]. The regulation of PSME1 and PSME2 ubiquitylation by HERC2 also points to a role for this ubiquitin ligase in immunoproteasome assembly and antigen processing [11]. Altogether, these results emphasize the key role of HERC2 in proteasome assembly machinery.

### HERC2 interacts with and regulates the stability of 19S regulatory particle subunits

**Fig. 3:**
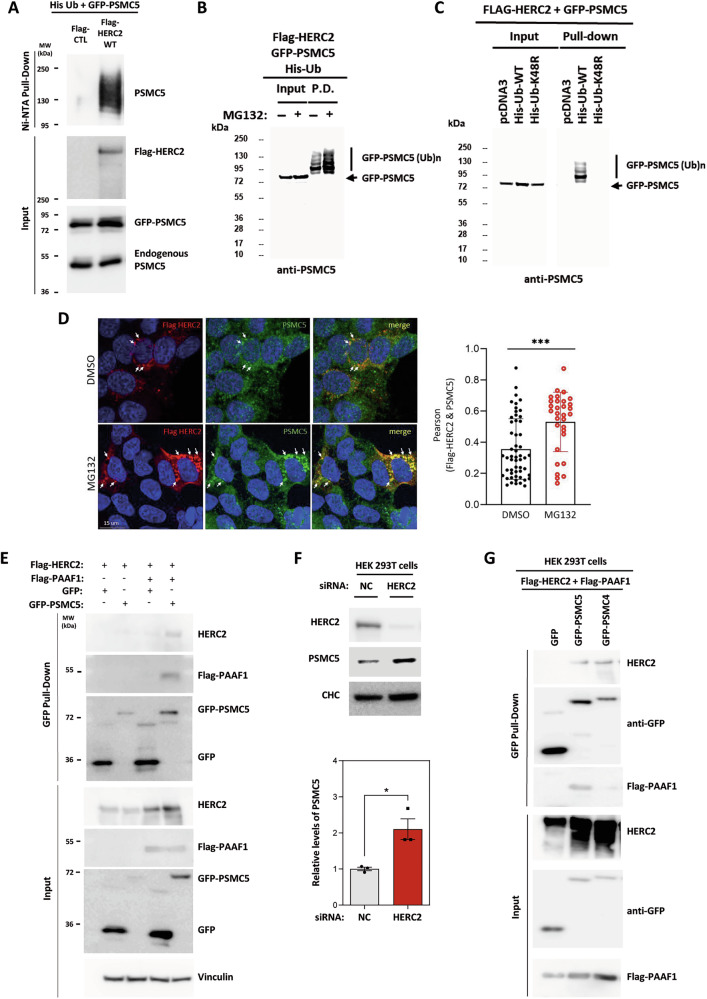
HERC2 regulates PSMC5 ubiquitylation and stability. **A** HEK-293T cells were transfected with His-Ub, GFP-PSMC5, and either a control plasmid (Flag-CTL) or the wild-type Flag-HERC2 (WT). After 48 h, the cells were treated with proteasome inhibitor MG132 (10 μM) for 6 h. Next, cell lysates were subjected to pull-down using Ni-NTA agarose. Inputs and proteins retained in the resin were analyzed by immunoblotting with antibodies against the indicated proteins. **B** HEK-293T cells were treated and analyzed as in (**A**). **C** HEK-293T cells were transfected with Flag-HERC2, GFP-PSMC5, and either a control plasmid (pcDNA3), the wild-type His-Ub (WT) or the mutant His-Ub K48R. After 48 h, the cells were treated with proteasome inhibitor MG132 (10 μM) for 4 h. Next, cell lysates were subjected to pull-down using GFP-trap resin. Inputs and proteins retained in the resin were analyzed by immunoblotting with antibodies against PSMC5. **D** (Left) Representative confocal images (maximum intensity projections) of HEK-293T cells transfected with Flag-HERC2 and incubated with either DMSO or MG132, followed by immunostaining with antibodies against PSMC5 and Flag. Scale bars, 15 μm. (Right) Quantification of co-localization between Flag-HERC2 and endogenous PSMC5, expressed as Pearson’s correlation coefficient. Data represent mean ± SEM from 54 images of DMSO-treated cells and 31 images of MG132-treated cells across 4-6 independent experiments. ***p < 0.001, unpaired Student’s *t* test. **E** HEK-293T cells were transfected with the indicated constructs. After 48 h, cell lysates were subjected to pull-down using GFP-trap resin. Inputs and proteins retained in the resin were analyzed by immunoblotting with antibodies against the indicated proteins. **F** HEK-293T cells were transfected with non-targeting control (NC) or HERC2 siRNAs for 72 h. Cell lysates were analyzed by immunoblotting with antibodies against the indicated proteins. PSMC5 protein levels were quantified and normalized based on clathrin heavy chain (CHC) protein levels. The plot represents the mean ± standard error of the mean. Representative results are shown from experiments repeated three times, and the individual data points are plotted as single dots. Significance levels: * *p* ≤ 0.05. **G** HEK-293T cells were transfected with the indicated constructs. After 48 h, cell lysates were subjected to pull-down using GFP-trap resin. Inputs and proteins retained in the resin were analyzed by immunoblotting with antibodies against the indicated proteins.

To investigate the molecular mechanisms involved in HERC2’s regulation of ubiquitylation of 19S assembly proteins, we focused on one of the subunits, PSMC5, and the chaperone PAAF1 involved in its assembly [12]. We demonstrated that PSMC5 ubiquitylation depends on HERC2 expression (Fig. 3A). Proteasome inhibition with MG132 increased PSMC5 ubiquitylation (Fig. 3B), which was associated with K48-linked polyubiquitin chains (Fig. 3C), indicating targeting for degradation. Confocal microscopy confirmed colocalization of HERC2 and PSMC5, which was enhanced upon proteasome inhibition (Fig. 3D). Pull-down assays validated the interaction between HERC2 and PSMC5, and revealed that this interaction requires the presence of PAAF1 (Fig. 3E). Notably, siRNA-mediated knockdown of HERC2 led to increased PSMC5 stability, supporting the role of HERC2 in regulating PSMC5 levels through ubiquitylation and proteasomal degradation (Fig. 3F).

Our proteomic analysis identified several 19S regulatory particle subunits that could interact with HERC2 in a manner similar to PSMC5. To explore this possibility, we investigated the interaction between HERC2 and another base subunit, PSMC4. Pull-down experiments demonstrated the interaction between HERC2 and PSMC4 independently of PAAF1 binding (Fig. 3G), suggesting that HERC2 may broadly engage with multiple components of the 19S assembly machinery.

### Unassembled subunits are regulated by HERC2

**Fig. 4:**
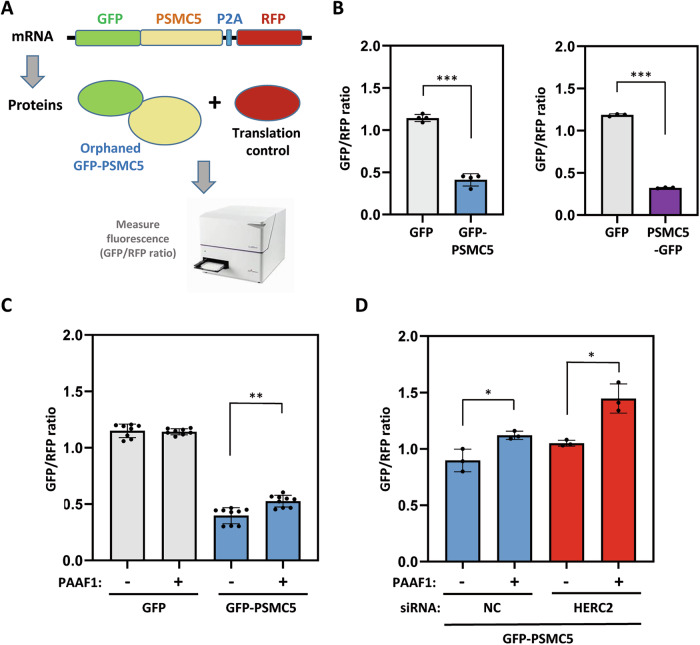
HERC2 regulates degradation of unassembled PSMC5 subunits. **A** Fluorescent reporter to monitor unassembled PSMC5 stability. Construct encodes PSMC5 fused to GFP in-line with red fluorescent protein (RFP) and separated by a P2A ribosome skipping sequence. **B** HEK-293T cells were transfected with fluorescent reporters expressing GFP-PSMC5 and RFP (indicated as GFP-PSMC5), or expressing PSMC5-GFP and RFP (indicated as PSMC5-GFP). A fluorescent reporter expressing GFP and RFP (indicated as GFP) was used as a control. After 48h, GFP and RFP fluorescence from cell lysates was measured in triplicate. GFP/RFP ratio is shown. Plots represent mean ± standard error of the mean. Experiments were repeated at least three times, and the data points of each experimental repetition are plotted as single dots. Significance levels: * *p* ≤ 0.05; ** *p* ≤ 0.01; *** *p* ≤ 0.001. **C** HEK-293T cells were transfected with the above fluorescent reporters GFP-PSMC5 or GFP together with constructs expressing Flag-PAAF1 (+) or plasmid control (–) as indicated. GFP/RFP ratio was measured and analyzed as in (**B**). **D** HEK-293T cells were transfected with non-targeting control (NC) or HERC2 siRNAs. After 24 h, these cells were transfected with the fluorescent reporter GFP-PSMC5 together with constructs expressing Flag-PAAF1 (+) or plasmid control (–) as indicated. 48 h later, the GFP/RFP ratio was measured and analyzed as in (**B**).

To delve deeper into the HERC2-regulated assembly mechanism, we used PSMC5 fused to GFP as a fluorescent reporter and an in-line red fluorescent protein (RFP) control separated by a P2A ribosome skipping sequence (Fig. 4A). The GFP/RFP ratio offers a quantitative measure of changes in the stability of the GFP-tagged protein. As expected, the fusion of PSMC5 to GFP destabilized GFP, with GFP-PSMC5 being observed to degrade more rapidly than GFP alone (Fig. 4B and Supplementary Fig. 2A). This destabilization was independent of GFP positioning, as both N- and C-terminal GFP fusions behaved similarly (Fig. 4B). Interestingly, PAAF1 overexpression specifically stabilized the GFP-PSMC5 reporter without affecting the GFP control (Fig. 4C). Conversely, HERC2 depletion stabilized the PSMC5 reporter (Fig. 4D and Supplementary Fig. 2B). Since PAAF1 detaches from PSMC5 once assembly is complete, the PAAF1-PSMC5 association indicates that only unassembled PSMC5 is a target for HERC2. Thus, prolonged association of PAAF1 with PSMC5 shows that assembly has failed, resulting in the recognition and degradation of unassembled PSMC5 by HERC2 (Fig. 4D).

### HERC2 regulates proteasome activity

**Fig. 5:**
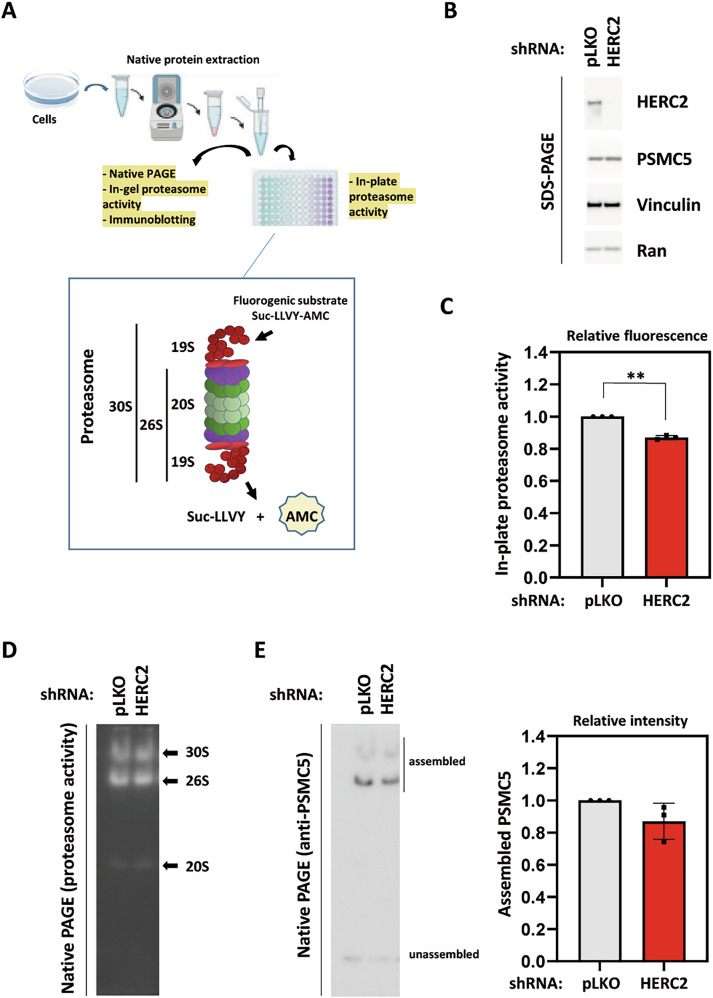
HERC2 regulates proteasome activity. **A** Strategy to analyze proteasome activity using the fluorogenic substrate Suc-LLVY-AMC. **B** Stable HEK-293T cell lines expressing a HERC2 shRNA and a non-targeting shRNA as control (pLKO) were generated. Lysates were analyzed by SDS-PAGE and immunoblotting with antibodies against the indicated proteins. A representative immunoblot is shown. **C** Proteasome activity was measured in stable HEK-293T cell lines expressing a HERC2 shRNA and a non-targeting shRNA (pLKO). Cells were lysed under native conditions. After centrifugation, supernatants were incubated with the proteasome substrate on a plate, and their fluorescence was measured in triplicate. Fluorescence was normalized by protein concentration and expressed relative to the control. Plots represent mean ± standard error of the mean. The experiment was repeated at least three times, and the data points of each experimental repetition are plotted as single dots. Significance levels: ** *p* ≤ 0.01. **D** Functional analysis of proteasome. Supernatants, prepared as in (**C**), were analyzed in native PAGE gels. After electrophoresis, the gel was incubated with the proteasome substrate and proteasome activity was measured. 30S, 26S and 20S structures are indicated. **E** Supernatants were prepared and analyzed in native PAGE gels as described in (**D**). Next, immunoblotting with anti-PSMC5 antibodies was performed. Assembled and unassembled forms of PSMC5 are indicated. The experiment was repeated three times. Assembled PSMC5 levels were quantified and expressed as relative intensity with respect to the control.

By controlling the ubiquitylation of base, lid, and 19S assembly chaperone proteins, HERC2 may play a central role in proteasome function. To analyze proteasome activity, we used the fluorogenic substrate, Suc-LLVY-AMC (Fig. 5A). Chronic HERC2 depletion (Fig. 5B) significantly decreased proteasomal activity (Fig. 5C). Under these conditions, the proteasome maintains its functional structure (30S and 26S), as observed by analyzing proteasomal activity in native gels (Fig. 5D). Interestingly, PSMC5 concentration did not vary with HERC2 depletion (Fig. 5B), probably because other cellular mechanisms affecting PSMC5 proteostasis are at play. Instead, the decrease in proteasomal activity due to chronic HERC2 depletion could be explained by a decrease in the quantity of proteasomes. Indeed, analysis of the assembled forms of PSMC5 present in the proteasome structure suggests this decrease (Fig. 5E). The impact of HERC2 loss on proteasomal activity extends beyond HEK-293T cells. MCF7 cells show similar vulnerability. In this cell line, the proteosome activity drops under HERC2 depletion (Supplementary Fig. 3). Taken together, these data establish HERC2 as a key regulator of proteasome activity and reveal a cell-type-dependent sensitivity to HERC2 loss. The greater vulnerability of MCF7 cells is likely related to their aneuploidy, which affects the balanced availability of subunits for efficient proteasome formation [12].

### Pathologenic HERC2 variant alters proteasome activity

**Fig. 6:**
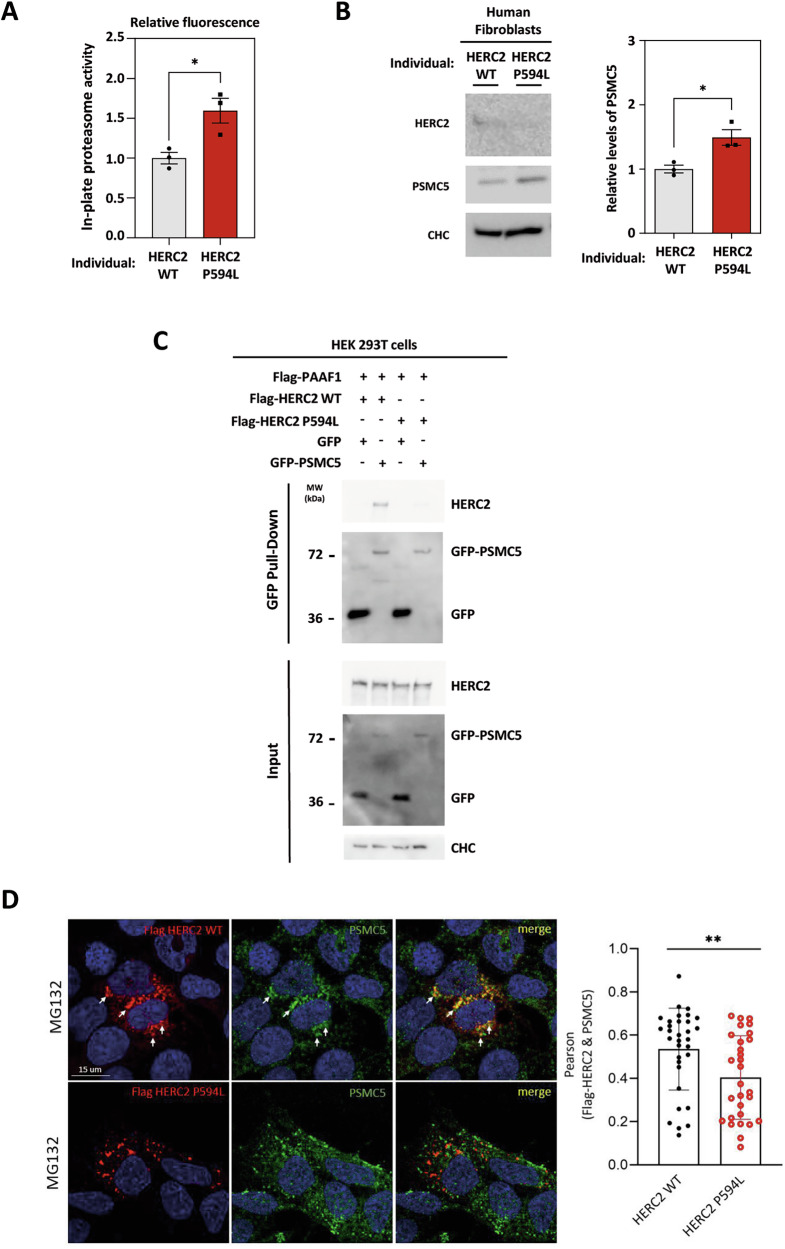
Pathologenic HERC2 variant alters proteasome activity. **A** Proteasome activity was measured on lysates from skin fibroblasts derived from three control individuals with the HERC2 WT variant and three patients with the HERC2 P594L variant. After centrifugation, supernatants were incubated with the proteasome substrate on a plate, and their fluorescence was measured in triplicate. Fluorescence was normalized by protein concentration and expressed relative to the control. Plots represent mean ± standard error of the mean. Significance levels: * *p* ≤ 0.05. **B** Lysates from skin fibroblasts derived from individuals with the HERC2 WT variant and patients with the HERC2 P594L variant were analyzed by immunoblotting with antibodies against the indicated proteins. PSMC5 protein levels were quantified and normalized based on clathrin heavy chain (CHC) protein levels. The plot represents the mean ± standard error of the mean. Significance levels: * *p* ≤ 0.05. **C** HEK-293T cells were transfected with the indicated constructs. After 48 h, cell lysates were subjected to pull-down using GFP-trap resin. Inputs and proteins retained in the resin were analyzed by immunoblotting with antibodies against the indicated proteins. **D** (Left) Representative confocal images (maximum intensity projections) of HEK-293T cells transfected with either Flag-HERC2 WT or P594L variant and treated with MG132. Cells were immunostained with anti-PSMC5 and anti-Flag antibodies. Scale bars, 15 μm. (Right) Quantification of co-localization between Flag-HERC2 WT or Flag-HERC2 P594L and endogenous PSMC5, measured using Pearson’s correlation coefficient. Data represent mean ± SEM from 32 images of Flag-HERC2 WT-transfected cells and 29 images of Flag-HERC2 P594L-transfected cells, acquired across 4 independent experiments. **p < 0.01, unpaired t-test.

Biallelic pathologenic variants in the *HERC2* gene, such as c.1781 C > T (p.Pro594Leu), cause a neurodevelopmental disorder clinically resembling Angelman syndrome. Understanding whether these pathologenic variants affect proteasome activity could help define potential new therapies for this disorder. To this end, we studied skin fibroblasts derived from individuals with the *HERC2 WT* and *HERC2 P594L* variant. Strikingly, proteasome activity is increased in cells derived from individuals with this mutation (Fig. 6A). Interestingly, and consistent with these data, a significant increase in PSMC5 protein levels was also observed, correlating with the reduced abundance of the unstable HERC2 P594L protein (Fig. 6B). To dissect the mechanism, we expressed a construct encoding the HERC2 P594L variant. Biochemical analysis showed that the HERC2 P594L protein exhibits poor interaction with the PSMC5-PAAF1 complex (Fig. 6C). Confocal imaging further confirmed these observations, demonstrating reduced colocalization between HERC2 P594L and PSMC5 proteins upon proteasome inhibition in the presence of MG132 (Fig. 6D). Together, all these results link pathologenic HERC2 variant with proteasome activity, shedding light on a molecular mechanism underlying the neurodevelopmental disorder it causes.

## Discussion

The experimental approach of combining the ^bio^Ub methodology with overexpression of the active (WT) and inactive (C4762S) forms of HERC2, and subsequent quantitative proteomic analysis of ubiquitylated proteins, has allowed us to identify 170 proteins regulated by ubiquitylation by this ubiquitin ligase. Most of these proteins are previously unknown substrates of HERC2, so their identification represents a major advance in our understanding of the molecular mechanisms that regulate them. Functional analysis of the identified proteins has allowed us to detect cellular processes regulated by HERC2. Nine processes stand out: proteasome complex, aminoacyl-tRNA synthetase multienzyme complex, translation initiation factor activity, AP-type membrane coat adaptor complex, Arp2/3 protein complex, centrosome, microtubule-associated complex, Ski complex, and GATOR2 complex. Although HERC2’s involvement in some of these processes, such as centrosome formation, was already known [13–15], its participation in others, such as t-RNA biosynthesis [16, 17], was completely unknown. Interestingly, the identification of the proteins regulated by HERC2 activity allowed us to observe how many of them were subunits of multiprotein complexes associated with these cellular processes, suggesting a role of HERC2 in their assembly. For example, in eukaryotic cells, aminoacyl-tRNA synthetases (ARSs) are categorized into two groups based on their association with the multi-tRNA synthetase complex (MSC): those that integrate into the MSC and those that remain free in the cytoplasm. The MSC is a large macromolecular complex comprising eight cytoplasmic ARSs and three non-enzymatic cofactors known as AIMPs (ARS-interacting multifunctional proteins), which mainly play scaffolding roles in the assembly [16, 17]. Notably, the E3 ubiquitin ligase HERC2 regulates the ubiquitination of five MSC components: EPRS, IARS, MARS, QARS, and AIMP1 (Table 1).

Functional analysis of the proteomic study highlighted the proteasome as one of the most important cellular processes regulated by HERC2. This analysis allowed us to identify numerous subunits involved in proteasome assembly and regulated by ubiquitylation by HERC2. Of the two particles that constitute the proteasome, the catalytic particle and the regulatory particle, HERC2 regulates the ubiquitylation of at least eleven proteins involved in the assembly of the regulatory particle. Nine proteins are related to the ATP-dependent 19S regulatory particle. PSMC1, PSMC2, PSMC4 and PSMC5 are AAA^+^-ATPases that are part of the heterohexameric ring that forms the base of the 19S regulatory particle, which binds directly to the 20S core particle of the proteasome. PSMD1 and PSMD2 are non-ATPases that bind to the heterohexameric ring, also forming the base structure [18]. PSMD3 is part of the lid of the 19S regulatory particle [19]. PSMD10 and PAAF1 are base assembly chaperones. These two chaperones are not present in the structure of the fully assembled proteasome but are essential for the regulatory particle assembly pathway. Each chaperone binds to the carboxy-terminal domain of a specific PSMC protein, aiding and accompanying its assembly. Specifically, PSMD10 binds PSMC4, and PAAF1 binds PSMC5 [20]. The other two proteins regulated by HERC2 involved in the assembly of the regulatory particle are PSME1 and PSME2 (also known as PA28α and PA28β, respectively). These two proteins form an ATP-independent 11S regulatory particle, which, together with a catalytic particle, forms the immunoproteasome, whose main function is the processing of class I MHC peptides [21]. The proteolytic activity of the proteasome depends on its abundance, which is controlled at the level of its subunit assembly [22]. Although future work will be necessary to fully understand the role of HERC2 in regulating the ubiquitylation of each of the identified subunits in the assembly of the regulatory particle, the studies performed in this work involving the subunits PSMC5 and PSMC4, and the chaperone PAAF1, indicate a way forward. Our data reveal an interaction of HERC2 with PSMC5 that is dependent on the chaperone PAAF1 and that shapes PSMC5 stability. Moreover, the identification of numerous HERC2-ubiquitylated proteins that belong to larger macromolecular complexes suggests that the function of HERC2 may extend broadly to the regulation of subunit assembly within these complexes. Consistent with this idea, HERC2 has previously been implicated in the assembly of subunits of the cytosolic chaperonin CCT, where it recognizes unassembled CCT subunits using ZNRD2 as an adaptor and tagging them with ubiquitin for degradation [23].

Assembly quality control pathways are essential guardians of proteostasis and cell viability, and their importance becomes especially apparent in long-lived, post-mitotic cells such as neurons, which rely on these mechanisms to prevent age-related proteotoxic stress [24]. Consistent with this, neuronal deficits frequently emerge when large HERC proteins are perturbed [2]. In mice, the loss of one allele of *HERC2* produces altered proteostasis in Purkinje cells, leading to loss of these cerebellar neurons and motor coordination defects [25]. Severe neurodegeneration is observed in the so-called *tambaleante* mouse, which expresses a *HERC1* variant deficient in interaction with PAAF1 [12, 26]. In humans, biallelic hypomorphic variants of *HERC2* are linked with neurodevelopmental disorders, which clinically resemble Angelman syndrome and are characterized by global developmental delay, intellectual disability, autism, and movement disorders [5–9, 12, 27]. Our work provides mechanistic insight into this connection: fibroblasts derived from individuals carrying the common P594L pathogenic variant exhibit elevated PSMC5 stability and increased proteasome activity. Two mechanisms appear to drive this phenotype: (1) the mutant HERC2 P594L protein is more unstable, and (2) the mutant P594L variant weakens the interaction between HERC2 and the PSMC5-PAAF1 complex. At the structural level, the P594L mutation occurs on a highly conserved proline in the RLD1 domain of HERC2 [4]. Interestingly, the HERC1 G483E mutation associated with the *tambaleante* phenotype and which prevents interaction with the PSMC5-PAAF1 complex, occurs on a highly conserved glycine in the RLD1 domain of HERC1 [12]. RLD domains are one of the distinguishing structural features of the HERC protein family [28]. RLD domains have a seven-bladed β propeller structure. Glycines and prolines are highly conserved amino acids in these domains and are responsible for this structure. Substitution of these amino acids may result in a conformational change of the RLD domain affecting protein stability and its interaction with other proteins. In agreement, a shorter half-life has been shown for the HERC2 P594L protein [4] and an impairment of the interaction between the RLD1 domain of the HERC1 G483E protein and clathrin [29].

Our findings establish a link between HERC2-related neurodevelopmental disorder and impaired proteasome activity, elucidating the molecular mechanisms by which RLD-domain mutations destabilize HERC proteins, disrupt proteostasis, and contribute to human pathology. Furthermore, these insights may have significant implications for preventive medicine. Early genetic screening for HERC2 variants could help identify individuals at risk of developing this disorder. In such cases, it would be valuable to investigate whether the expected alterations could be mitigated or prevented by the use of proteasome inhibitors that restore the balance in proteasomal function.

## Materials and methods

### Human cell samples, cell culture and treatments

Samples of human skin fibroblasts were obtained with approved informed consent as previously described elsewhere [4].

HEK‑293 T, MCF7 and human skin fibroblasts were cultured as previously described [27]. Where indicated, cells were treated with MG132 (10 μM) for 3–6 h. Plasmid transfections were carried out using Lipofectamine LTX transfection reagent (Invitrogen, Carlsbad, CA) or Polyethylenimine (PEI) according to the manufacturer’s instructions. siRNA-mediated knockdown, shRNA-mediated knockdown, and lentiviral particle production and cell infection were performed as previously described [27].

### Constructs

pMD2.G (Addgene #12259) and psPAX2 (Addgene #12260) were a gift from Didier Trono. pLKO.1 - TRC control was a gift from David Root (Addgene #10879). His-Ub and His-Ub-K48R were a gift from Tatiana Erazo. KY28 (EGFP-P2A-mCherry), EZ103 (EGFP-PSMC5-P2A-mCherry), EZ144 (EGFP-PSMC4-P2A-mCherry), and EZ125 (3xFlag-PAAF1) plasmids were a gift from Ramanujan S. Hegde [12]. pcDNA5 FRT/TO SF-HERC2 (Addgene #55613) and pcDNA5 FRT/TO SF-HERC2 C4762S (Addgene # 55614) were a gift from David Chan. pcDNA5 FRT/TO SF-HERC2 P594L was generated from pcDNA5 FRT/TO SF-HERC2 using in vivo assembly by homologous recombination [30] and sequenced. pCAG-(^bio^Ub)x6-BirA was previously described [9].

### Protein extraction, SDS-PAGE, and immunoblotting

Protein extraction, SDS-PAGE, and immunoblotting were performed as previously described [27]. Antibodies used: anti-HERC2 (BD Biosciences, 610151); anti-clathrin heavy chain (Santa Cruz Biotechnology, sc12734)**;** anti-Flag M2 (Sigma-Aldrich, F3165 and F7425)**;** anti-GFP (Abcam, ab13970)**;** anti-PSMC5 (Santa Cruz Biotechnology, sc390631); anti-α-tubulin (Abcam, ab7291); anti-vinculin (Santa Cruz Biotechnology, sc73614)**;** anti-Ran [31].

### Pull-down assay

To analyze protein-protein interactions, GFP pull-down assays were performed as previously described [12]. To analyze ubiquitylation, assays were performed as previously described [27]. Similar results were obtained using the urea lysis buffer (50 mM HEPES pH 7.4, 150 mM NaCl, 8 M urea, 1% CHAPS, 5 mM imidazole and 10 mM 2-mercaptoethanol) for lysis, incubation and washing.

### Immunocytochemistry and confocal imaging

Cells were analyzed as previously described [27] and using the antibodies indicated for immunoblotting. Antibodies conjugated to Alexa-Fluor 488, 568 or 647 were used as secondary antibodies.

### Quantitative proteomic analysis with the ^bio^Ub strategy

Biotin pull-downs and proteomic analysis were performed in triplicate as previously described [9]. See details in supplementary information. The mass spectrometry results have been deposited to the ProteomeXchange Consortium [32] with the dataset identifier PXD071427.

### Functional connectivity networks

Proteins with significantly increased ubiquitylation in HERC2 WT were analyzed using STRING (v.12.0) [33].

### Fluorescent reporter analysis

GFP and RFP fluorescence from lysates of cells transfected with fluorescent reporter constructs was measured using a CLARIOstar Plus microplate reader (BMG LABTECH, Ortenberg, Deutschland). See specific details in supplementary information. For flow cytometry analysis, we followed the protocol previously described [12].

### Proteasome activity and native PAGE

For proteasome activity in 96-well plates, equal amounts of protein (5–20 µg) were loaded in triplicate in 96-well dark plates containing 200 μL/well of Proteasome buffer supplemented with 50μM Suc-LLVY-AMC (Sigma-Aldrich, S6510), and incubated for 60 min at 37 °C in the dark. After this time, fluorescence was measured using a CLARIOstar Plus microplate reader (BMG LABTECH, Ortenberg, Deutschland). For proteasome activity in native PAGE, equal amounts of protein (5-20 µg) were loaded in 3-10% Tris-Acetate PAGE and run with Native gel buffer. After electrophoresis, the gel was washed and incubated with 50μM Suc-LLVY-AMC. After this time, a fluorescence image from the gel was captured using a UV Transilluminator (Bio-Rad, Hercules, CA). After proteasome activity, immunoblotting analysis was performed. See specific details in supplementary information.

### Statistical analysis

Data were analyzed using GraphPad Prism version 8.4.3 and expressed as means and standard error of the mean ( ± SEM) of at least three independent experiments. Individual data points are plotted as single dots. Significance was calculated by unpaired Student’s *t* tests and indicated as follows: *, **, or *** for *p*-values of ≤ 0.05, ≤ 0.01, or ≤ 0.001, respectively.

## Supplementary information

Supplementary Figure 1

Supplementary Figure 2

Supplementary Figure 3

Supplementary materials and methods

Supplementary Table 1

Raw data WB

## Data Availability

The mass spectrometry results have been deposited to the ProteomeXchange Consortium via the PRIDE [32] partner repository with the dataset identifier PXD071427. Original images are available on raw image file. Any additional information reported in this paper is available from the lead contact upon request.
