# Supplementary figures and images for: Proteasome dysfunction underlies HERC2-linked neurodevelopmental disorder with Angelman-like clinical features
Source: Cell Death Discov. 2026 Apr 8;12:243. doi: 10.1038/s41420-026-03095-x (PMC13187139; doi:10.1038/s41420-026-03095-x)

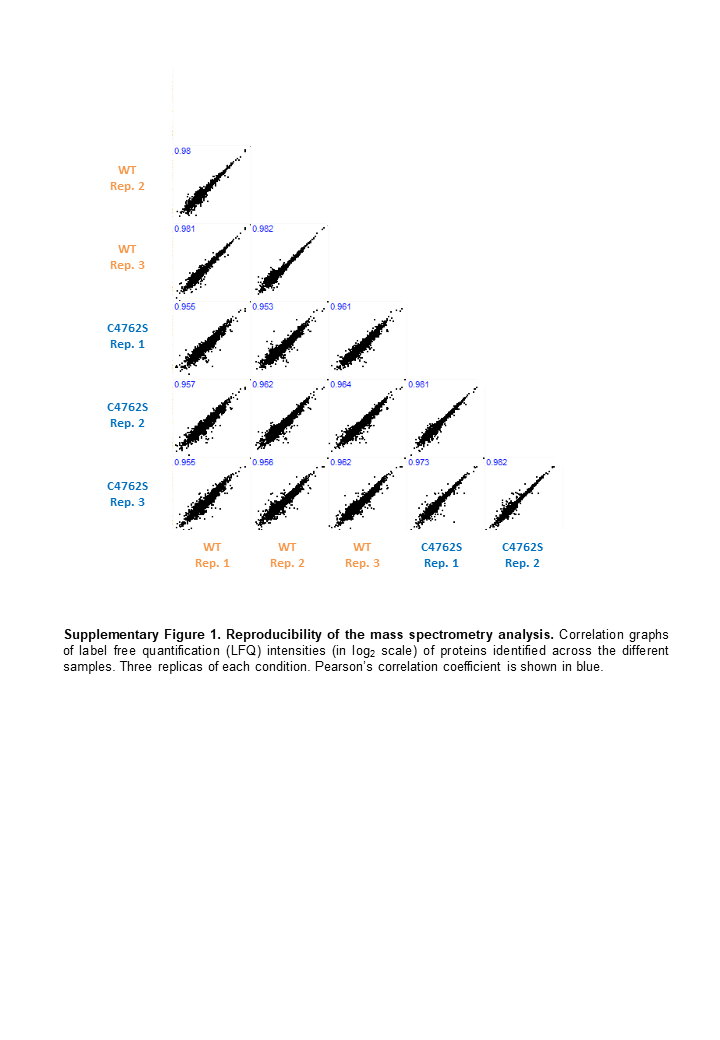

Supplement: Supplementary file 1 — Supplementary Figure 1 [file 41420_2026_3095_MOESM1_ESM.tif]

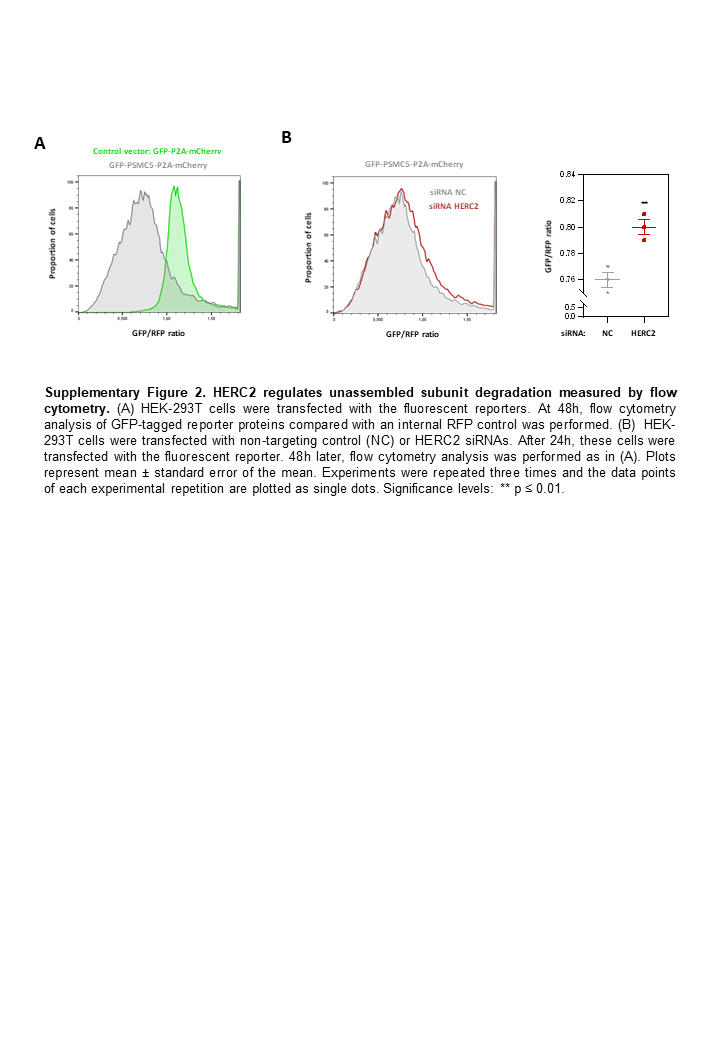

Supplement: Supplementary file 2 — Supplementary Figure 2 [file 41420_2026_3095_MOESM2_ESM.tif]

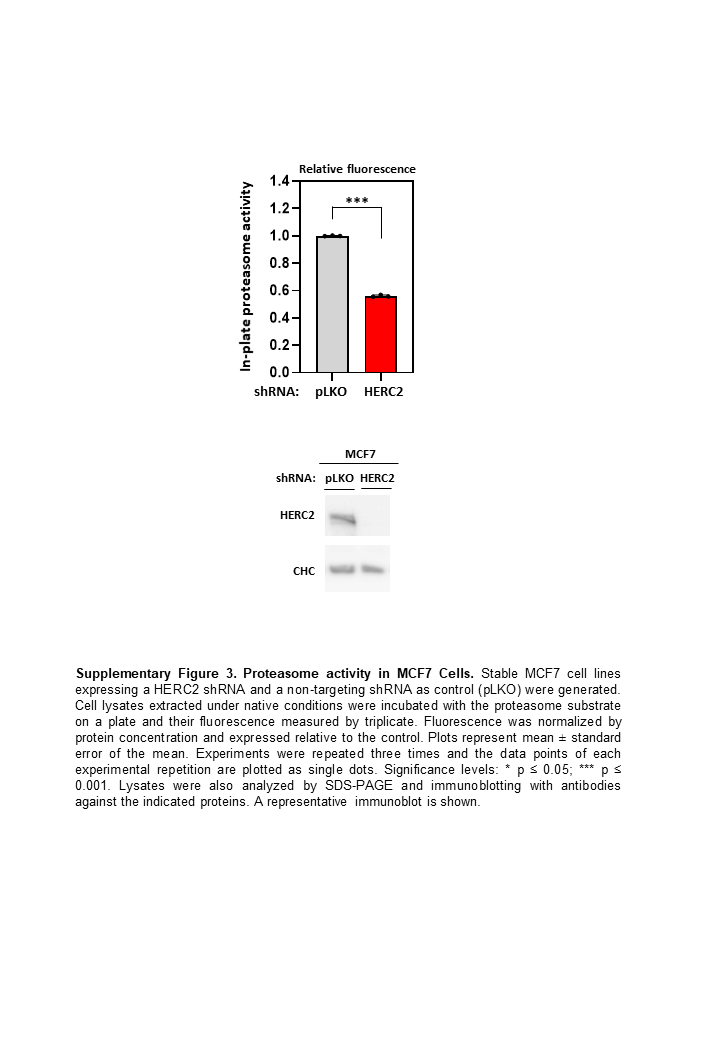

Supplement: Supplementary file 3 — Supplementary Figure 3 [file 41420_2026_3095_MOESM3_ESM.tif]
