# Supplementary material for: Proteasome dysfunction underlies HERC2-linked neurodevelopmental disorder with Angelman-like clinical features
Source: Cell Death Discov. 2026 Apr 8;12:243. doi: 10.1038/s41420-026-03095-x (PMC13187139; doi:10.1038/s41420-026-03095-x)
Supplement: Supplementary file 4 — Supplementary materials and methods [file 41420_2026_3095_MOESM4_ESM.docx]

**SUPPLEMENTARY INFORMATION**

**Proteasome dysfunction underlies HERC2-linked neurodevelopmental disorder with Angelman-like clinical features**

Joan Sala‑Gaston¹˒¹⁰, Laura Costa‑Sastre¹˒¹⁰, Manel Garcia‑Diez¹, Tania López‑Hernández¹, Juanma Ramírez², Nerea Osinalde², Jose Antonio Valer³, Claudia Arnedo‑Pac⁴, Bernat Crosas⁵, Emma L. Baple⁶, Andrew H. Crosby⁶, Ugo Mayor²˒⁷, Francesc Ventura¹, and Jose Luis Rosa¹˒⁸˒⁹*

¹ Department of Physiological Sciences, University of Barcelona, IDIBELL, Spain

² Department of Biochemistry and Molecular Biology, Faculty of Science and Technology, UPV/EHU, Leioa, Bizkaia, Spain

³ Cambridge Institute for Medical Research (CIMR), University of Cambridge, Cambridge, UK

⁴ MRC Toxicology Unit, University of Cambridge, Cambridge, UK

⁵ IBMB‑CSIC, Barcelona, Spain

⁶ University of Exeter Medical Research Centre, Exeter, UK

⁷ Ikerbasque, Basque Foundation for Science, Bilbao, Spain

⁸ Senior author; ⁹ Lead contact; ¹⁰ These authors contributed equally

*Correspondence: joseluisrosa@ub.edu

**SUPPLEMENTARY INFORMATION**

**MATERIALS AND METHODS**

**Quantitative proteomic analysis with the ^bio^Ub strategy**

Cell lysates were incubated with NeutrAvidin agarose beads. After washing with different buffers to reduce non-specific interactions, the ubiquitylated material was eluted from the beads. Eluted material was resolved by SDS-PAGE system. After the exclusion of avidin monomers and dimer, each lane was subjected to in-gel digestion as described previously ^1^. Briefly, digestion of proteins was performed by incubation of the gel slices with trypsin. The resulting peptides were extracted from the gel and subjected to LC-MS/MS analysis. Mass spectrometric analyses were performed on an EASY-nLC 1000 liquid chromatography system interfaced via a nanospray flex ion source with a Q Exactive (ThermoFisher Scientific, Waltham, MA, USA) mass spectrometer. Data were acquired using Xcalibur software (Thermo Fisher Scientific, Waltham, MA, USA).

MaxQuant output data was analyzed with Perseus (version 1.6.15.0) ^2^. See results in Supplementary Table 1. First, proteins only identified by site, contaminants, reverse hits and proteins with no unique peptides and/or no intensity were removed. Then, missing LFQ (label-free quantification) intensity values were replaced with values from a normal distribution (width 0.3 and down shift 1.8), meant to simulate expression levels below the detection limit. Statistically significant differences in protein abundance were determined by two-tailed Student’s t-test. Proteins considered to be more ubiquitinated in HERC2 WT samples should fulfill the following criteria: (1) display a LFQ fold change (WT vs C4762S) above 1 (in log_2_ scale) that is statistically significant (p < 0.05); (2) be detected by at least two unique peptides; and (3) contain no imputed values in any of the three replicas of at least one of the conditions, or have a maximum of one imputed value in each condition. The same criteria were applied for those proteins considered less ubiquitinated in HERC2 C4762S samples. The mass spectrometry results have been deposited to the ProteomeXchange Consortium^3^ with the dataset identifier PXD071427.

**Fluorescent reporter analysis**

Cells growing in 6-well plates were transfected with fluorescent reporter constructs using LTX transfection reagent (Invitrogen, Carlsbad, CA), according to the manufacturer’s instructions. 48 hours later, cells were washed with PBS and lysed by scrapping after adding CHAPS lysis buffer (10 mM Tris–HCl, 100 mM NaCl, and 0.3% CHAPS detergent, pH 7.5). This buffer was supplemented with protease and phosphatase inhibitors (50 mM β-glycerophosphate, 50 mM sodium fluoride (NaF), 1 mM sodium vanadate, 1 mM phenylmethylsulphonyl fluoride (PMSF), 5 µg/mL leupeptin, 5 µg/mL aprotinin, 1 µg/mL pepstatin A, 100 µg/mL benzamidine, 1 µM E-64). Lysates were sonicated and maintained on ice under agitation for 10 min before of their centrifugation at 13,000×g at 4°C for 10 min. The protein concentration of the supernatants was measured. Equal amounts of protein (1-10 µg) were loaded by triplicated in 96-well dark plates containing 200 mL/well of CHAPS lysis buffer supplemented with 5 mM DTT. GFP and RFP fluorescence was measured using a CLARIOstar Plus microplate reader (BMG LABTECH, Ortenberg, Deutschland).

**Proteasome activity and native PAGE**

HEK-293T cells grown in 10 cm plates (60-90% confluency) were washed with PBS cold and lysed (500μL/plate) on ice with Proteasome buffer (50mM HEPES/NaOH pH=7.8, 10mM NaCl, 1.5mM MgCl_2_, 1mM EDTA, 1mM EGTA, 250mM Sucrose, 5mM DTT, and 2mM ATP-Mg) supplemented with 0.002% Digitonin for 15 min. Lysates were centrifuged at 16,000×g for 10 min at 4°C and the protein concentration of the supernatants was measured.

For proteasome activity in 96-well plates, equal amounts of protein (5-20 µg) were loaded by triplicated in 96-well dark plates containing 200 μL/well of Proteasome buffer supplemented with 50μM Suc-LLVY-AMC, and incubated for 60 min at 37ºC in dark. After this time, fluorescence was measured using a CLARIOstar Plus microplate reader (BMG LABTECH, Ortenberg, Deutschland).

For proteasome activity in native PAGE, equal amounts of protein (5-20 µg) were loaded in 3-10% Tris-Acetate PAGE and running with Native gel buffer (90mM Tris-Base, 90mM Boric Acid, 5mM MgCl_2_, 0,1mM EDTA, 0.5mM DTT, and 0.5mM ATP-Mg) at 4ºC for 3h at ΔV=150V (approx. 70 mA). After electrophoresis, the gel was washed with Reaction buffer (50mM Tris-HCl pH=7.5, 5mM MgCl_2_, 1mM ATP-Mg) for 5 min, and incubated for 20 minutes at 37°C with Reaction buffer supplemented with 50μM Suc-LLVY-AMC. After this time, fluorescence image from the gel was captured using an UV Transilluminator (Bio-Rad, Hercules, CA).

To analyze proteins in native PAGE, after proteasome activity, the gel was incubated with Denaturing buffer (2% SDS + 66mM Na_2_CO_3_ + 1.5% β-OH) for 10 min. Next was washed with Transfer buffer (20% Methanol, 25mM Bicine, 25mM Bis-Tris,1 mM EDTA, and 13 mM Sodium bisulphite) and transferred overnight (DV = 20V) to a PVDF membrane. After this, immunoblotting analysis was performed as indicated above for SDS-PAGE.

**References:**

1 Osinalde N, Sánchez-Quiles V, Akimov V, Blagoev B, Kratchmarova I. SILAC-based quantification of changes in protein tyrosine phosphorylation induced by Interleukin-2 (IL-2) and IL-15 in T-lymphocytes. *Data Brief* 2015; 5: 53–58.

2 Ramirez J, Prieto G, Olazabal-Herrero A, Borràs E, Fernandez-Vigo E, Alduntzin U *et al.* A Proteomic Approach for Systematic Mapping of Substrates of Human Deubiquitinating Enzymes. *International Journal of Molecular Sciences Article J Mol Sci* 2021. doi:10.3390/ijms22094851.

3 Perez-Riverol Y, Bandla C, Kundu DJ, Kamatchinathan S, Bai J, Hewapathirana S *et al.* The PRIDE database at 20 years: 2025 update. *Nucleic Acids Res* 2025; 53: D543–D553.
