# Supplementary material for: Proteasome dysfunction underlies HERC2-linked neurodevelopmental disorder with Angelman-like clinical features
Source: Cell Death Discov. 2026 Apr 8;12:243. doi: 10.1038/s41420-026-03095-x (PMC13187139; doi:10.1038/s41420-026-03095-x)
Supplement: Supplementary file 6 — Raw data WB [file 41420_2026_3095_MOESM6_ESM.pdf]

RAW DATA

Proteasome dysfunction underlies HERC2-linked neurodevelopmental disorder with Angelman-like clinical features

Joan Sala-Gaston[1,10], Laura Costa-Sastre[1,10], Manel Garcia-Diez[1], Tania López-Hernández[1], Juanma Ramírez[2], Nerea Osinalde[2], Jose Antonio Valer[3], Claudia Arnedo-Pac[4], Bernat Crosas[5], Emma L. Baple[6], Andrew H. Crosby[6], Ugo Mayor[2,7], Francesc Ventura[1], and Jose Luis Rosa[1,8,9]*

Figure 1

B

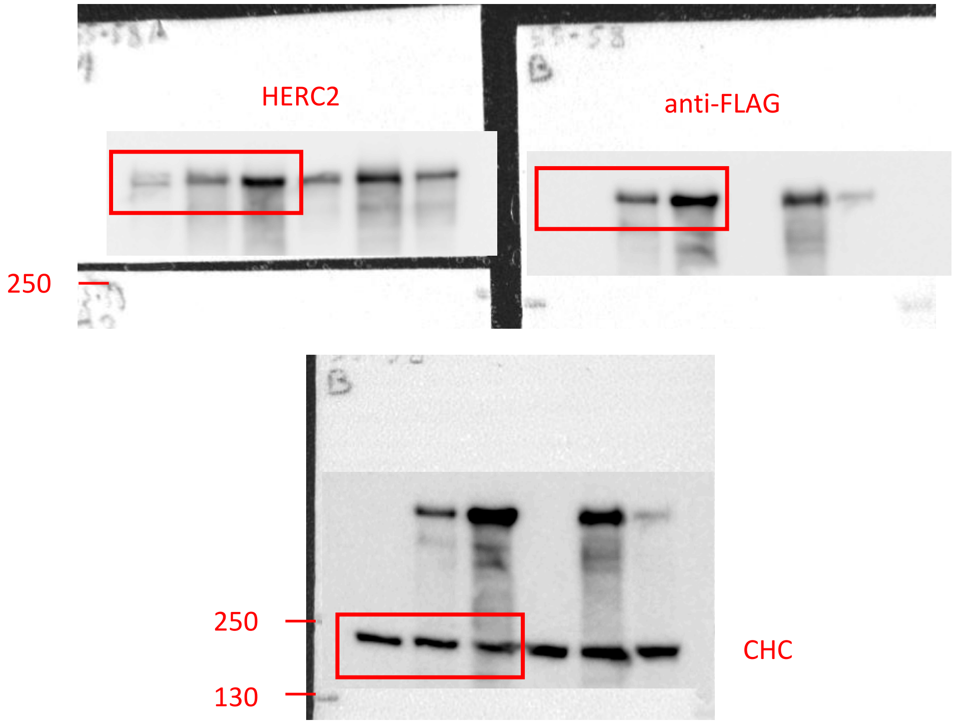
HERC2
anti-FLAG
250
250
130
CHC

C

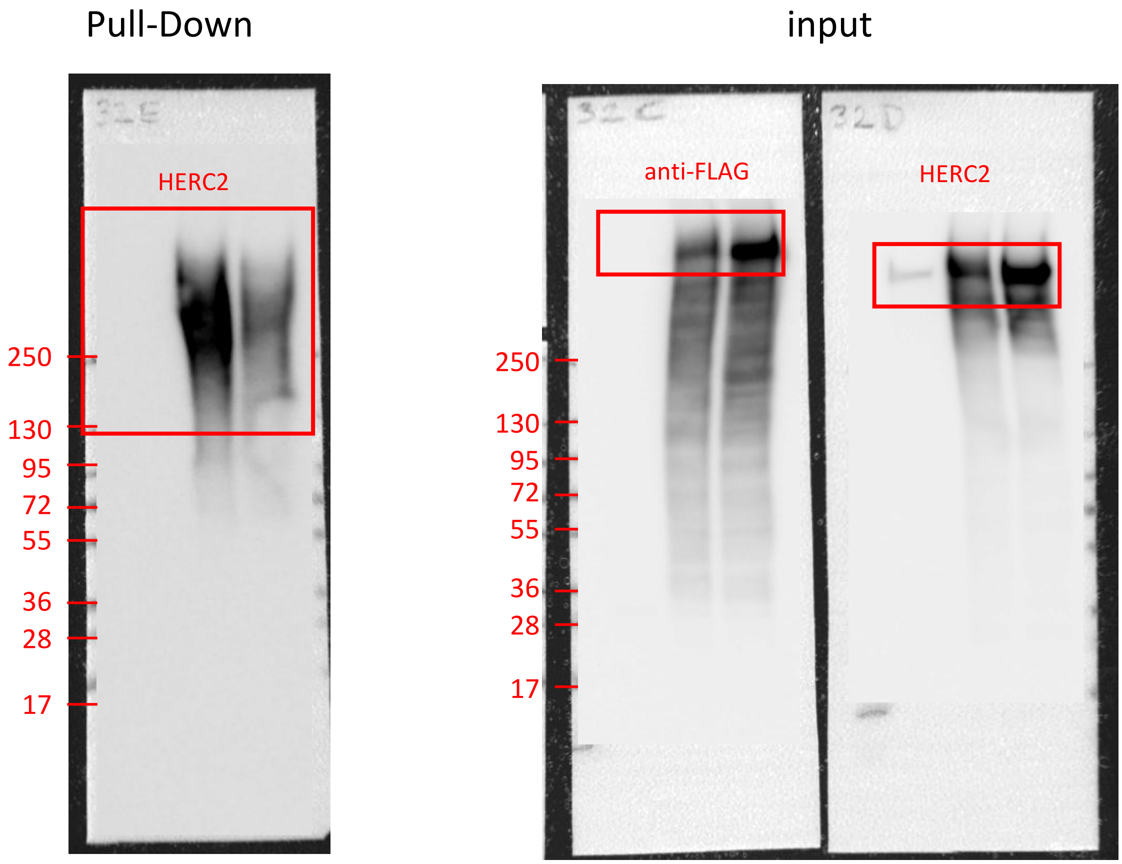
Pull-Down
input
HERC2
anti-FLAG
HERC2
250
130
95
72
55
36
28
17
250
130
95
72
55
36
28
17

Figure 3

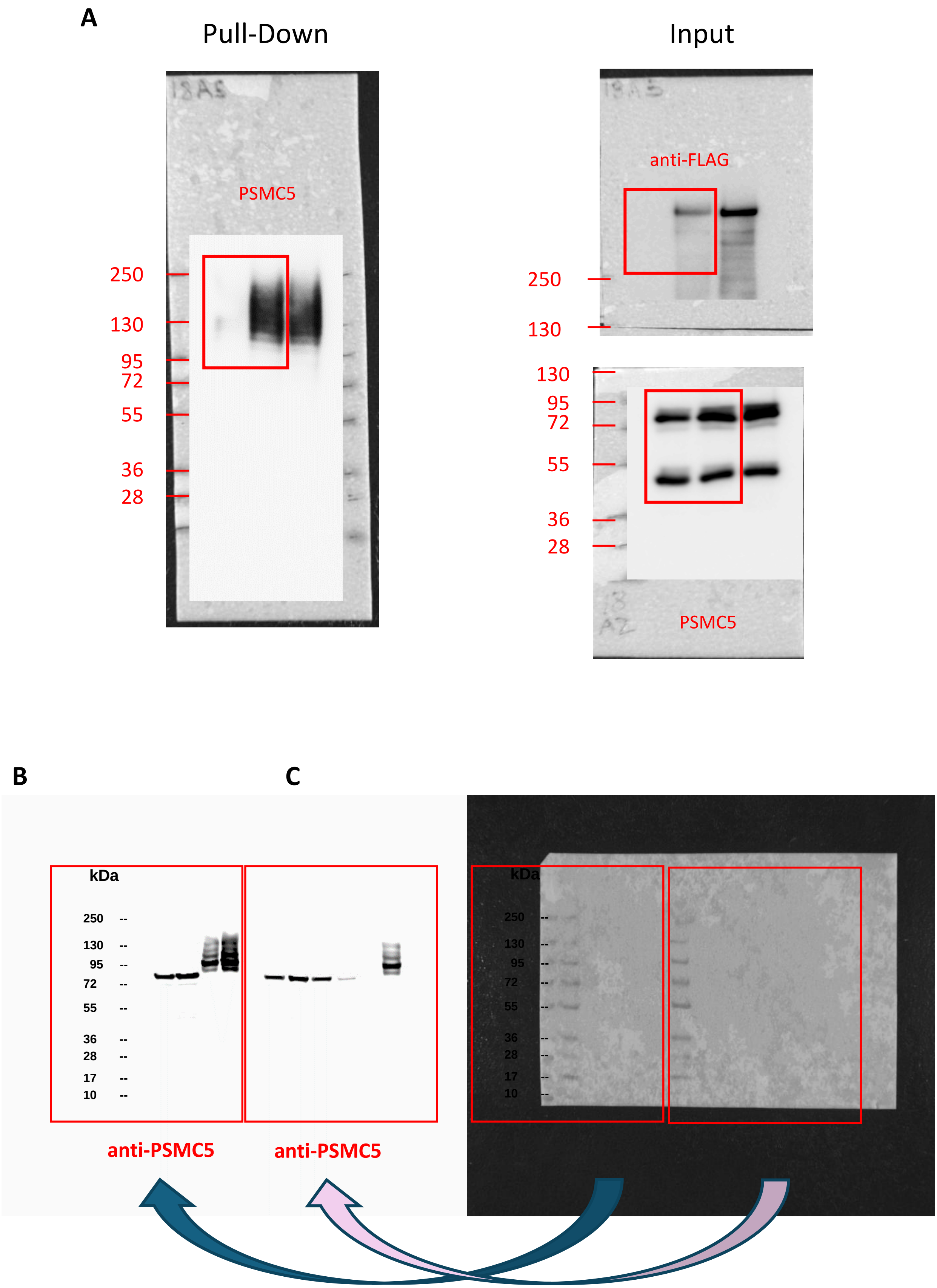
A
Pull-Down
Input
PSMC5
250
130
95
72
55
36
28
anti-FLAG
250
130
130
95
72
55
36
28
PSMC5
B
C
kDa
250
130
95
72
55
36
28
17
10
anti-PSMC5
anti-PSMC5
kDa
250
130
95
72
55
36
28
17
10

Figure 3

E

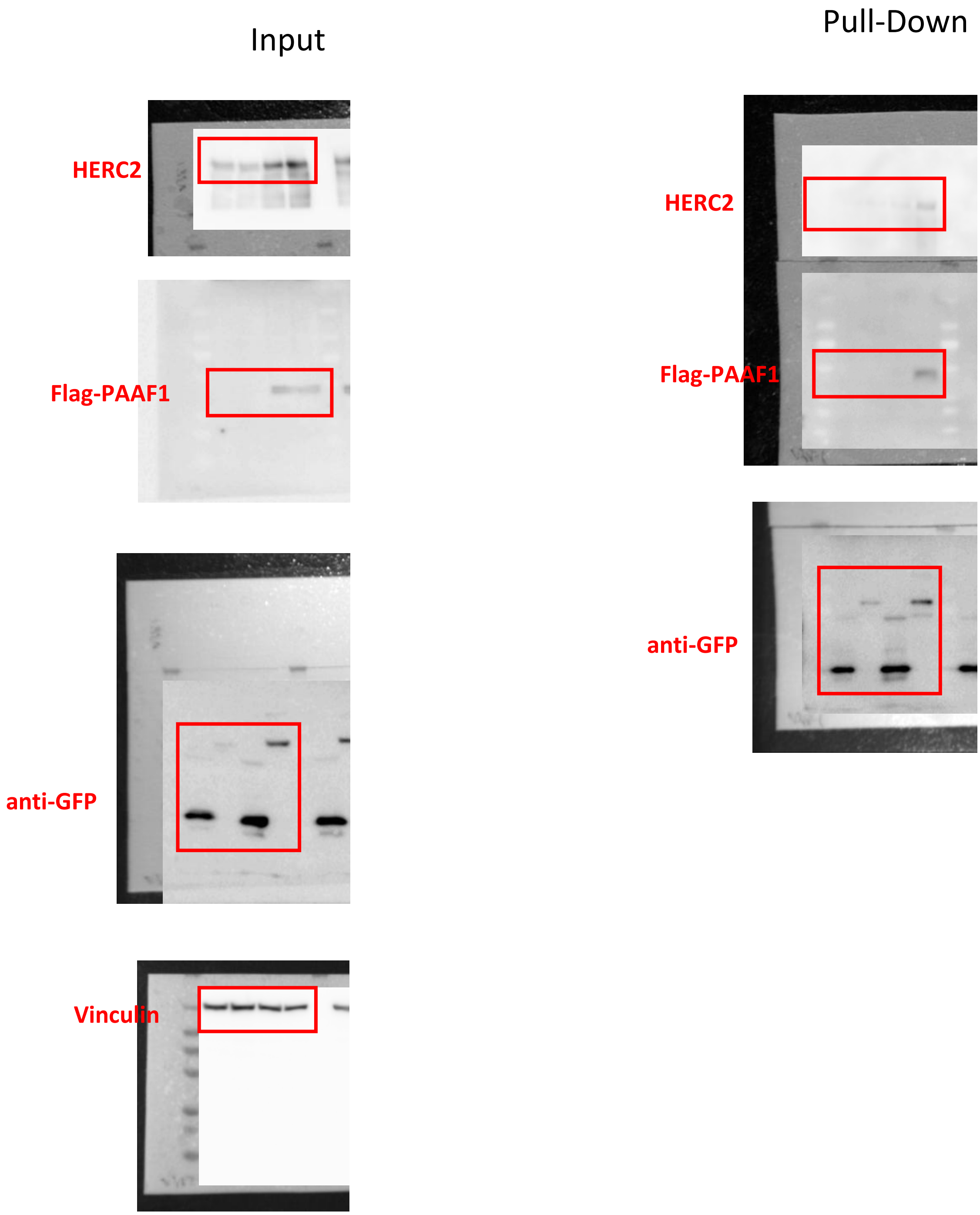
Input
Pull-Down
HERC2
Flag-PAAF1
anti-GFP
Vinculin
HERC2
Flag-PAAF1
anti-GFP

Figure 3

F

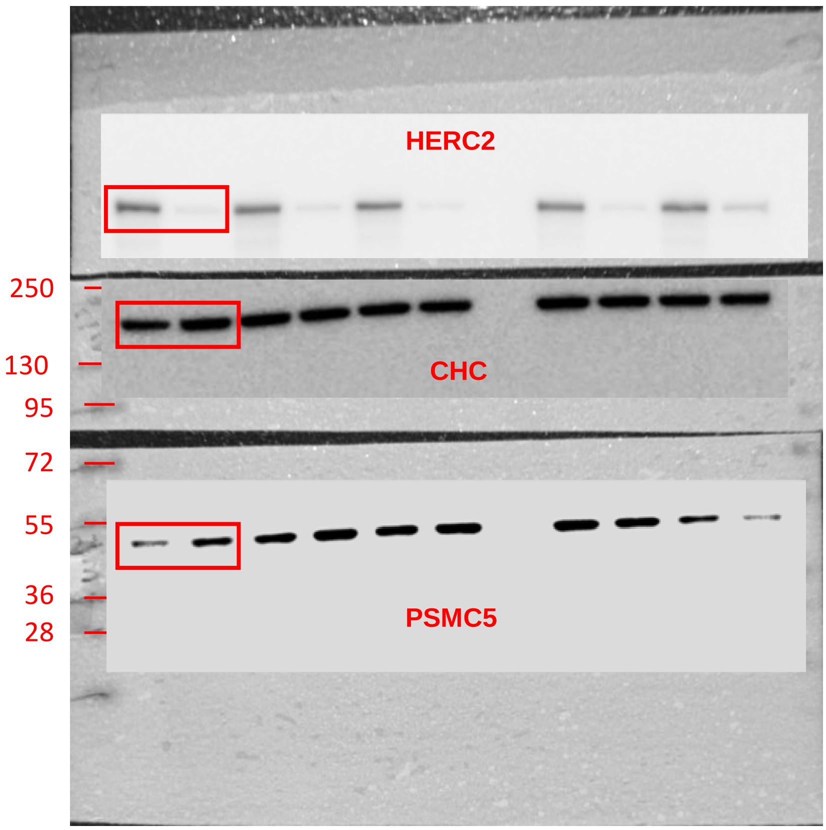
HERC2
250
130
95
72
55
36
28
CHC
PSMC5

G

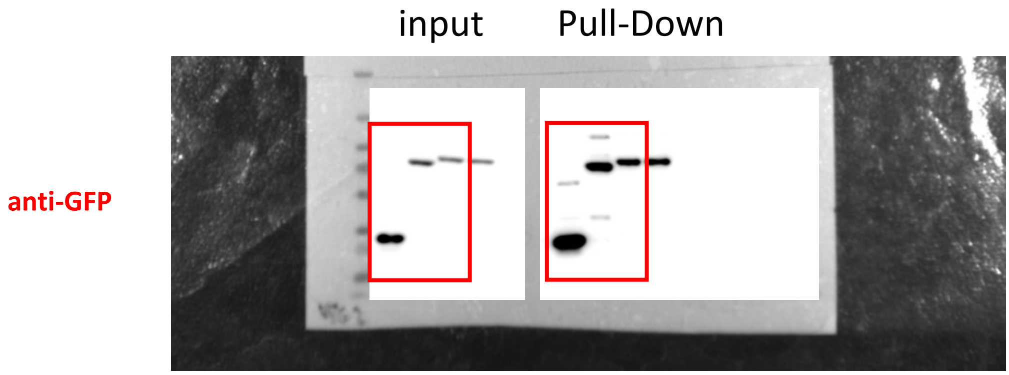
input
Pull-Down
anti-GFP

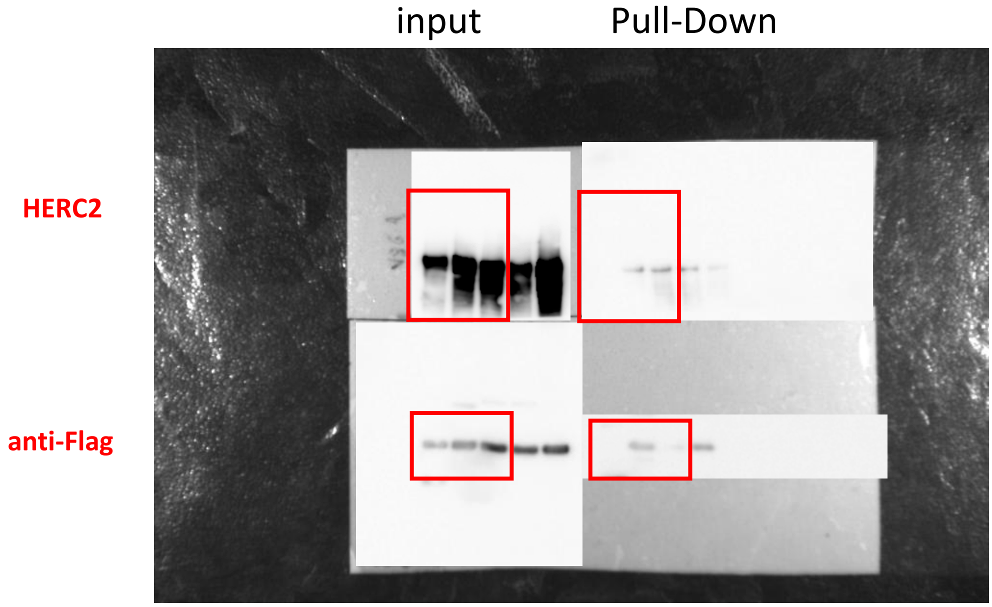
input
Pull-Down
HERC2
anti-Flag

Figure 5

B

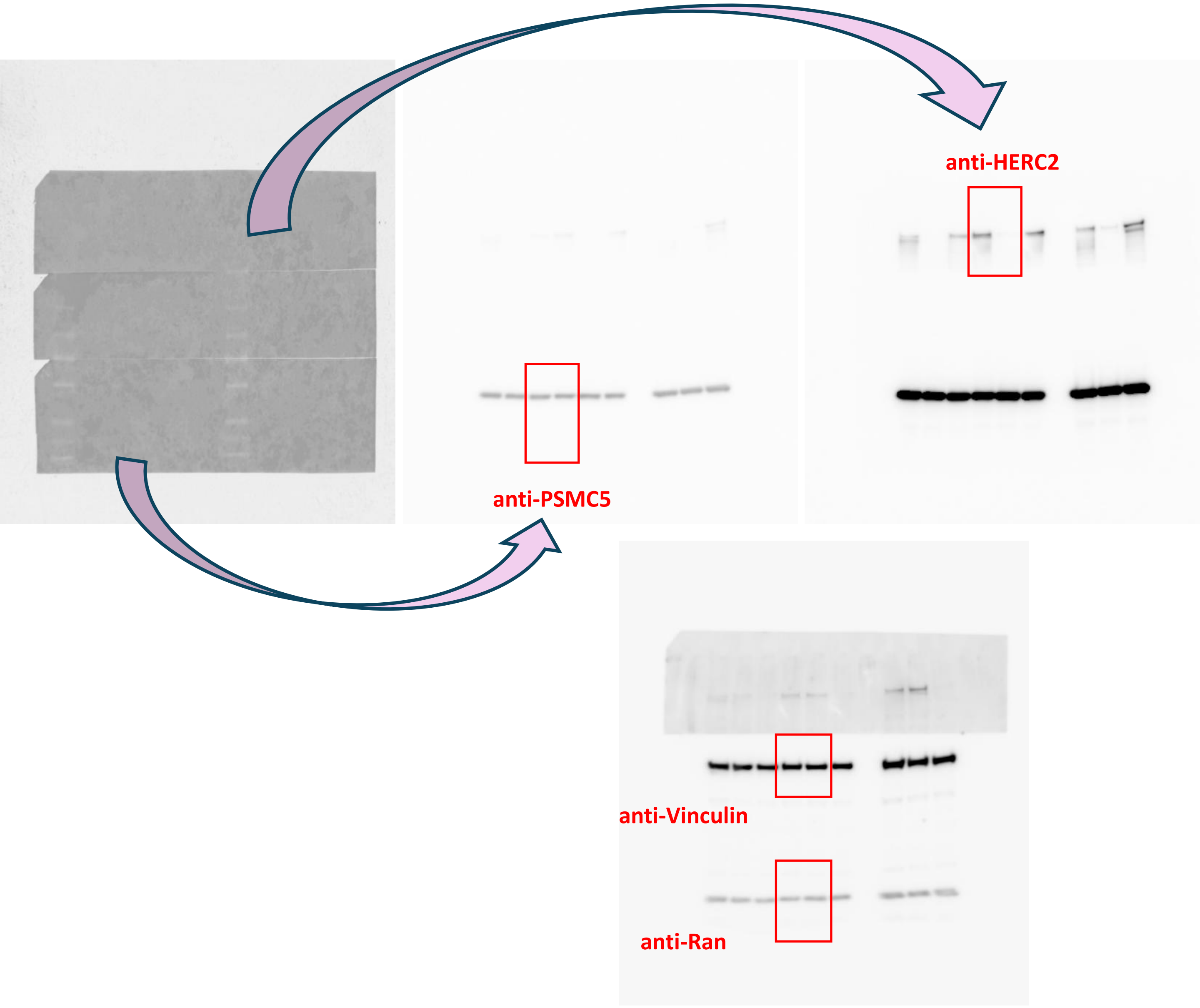

Figure 5

D

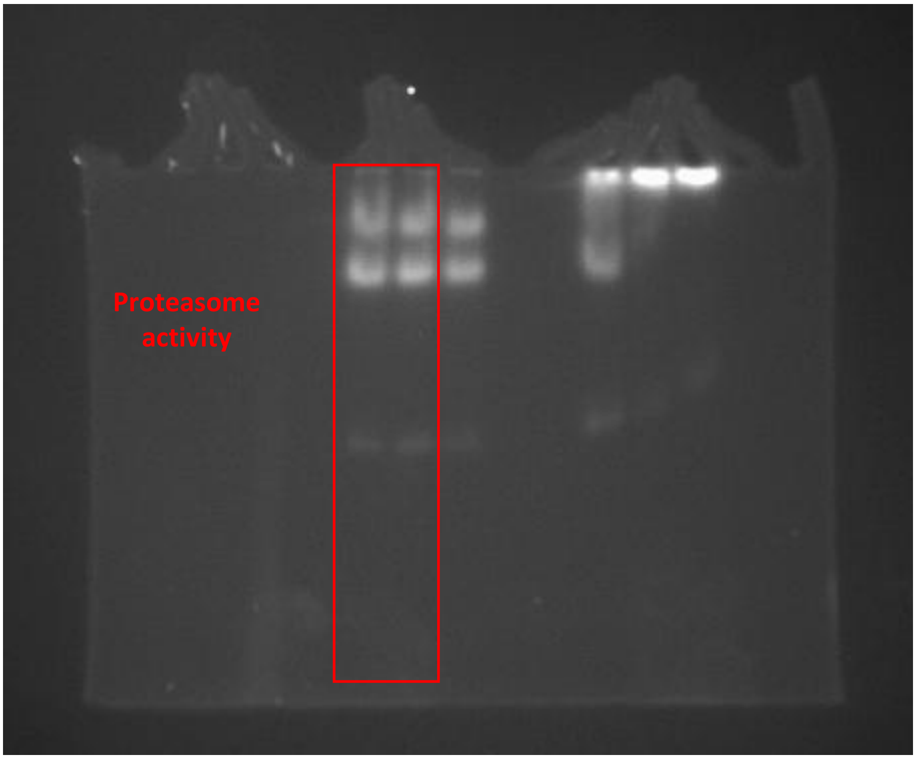

E

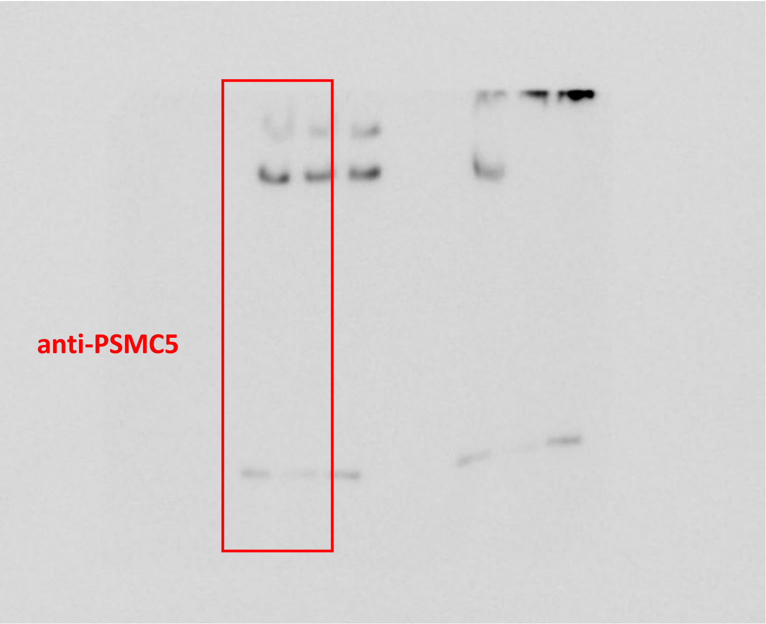

Figure 6

B

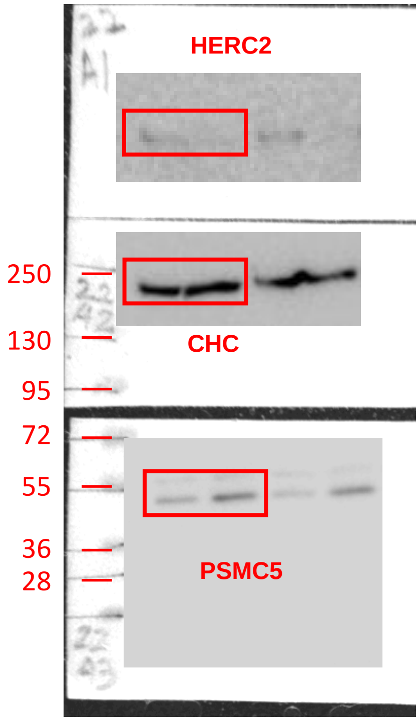
HERC2
250
130
95
72
55
36
28
CHC
PSMC5

C

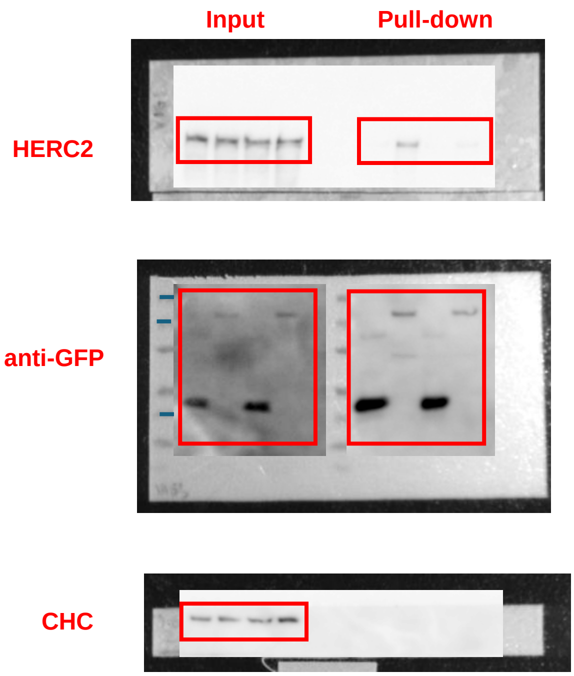
Input
Pull-down
HERC2
anti-GFP
CHC

Supplementary Figure 3

HERC2

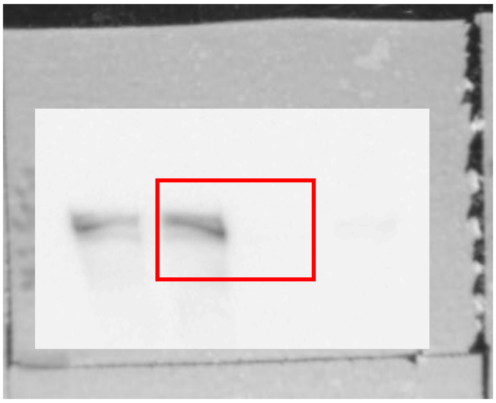

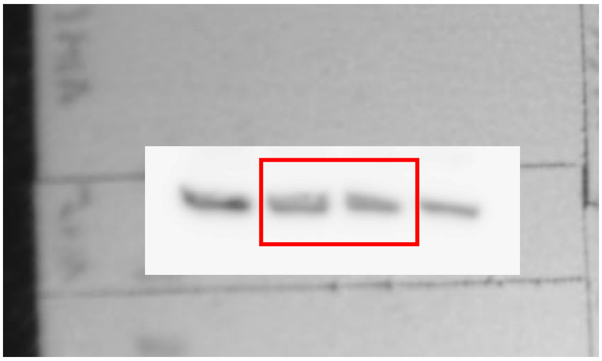

CHC
